# The contribution of industrial emissions to ozone pollution: identified using ozone formation path tracing approach

**DOI:** 10.1038/s41612-023-00366-7

**Published:** 2023-05-16

**Authors:** Junlei Zhan, Wei Ma, Boying Song, Zongcheng Wang, Xiaolei Bao, Hong-Bin Xie, Biwu Chu, Hong He, Tao Jiang, Yongchun Liu

**Affiliations:** 1 Aerosol and Haze Laboratory, Advanced Innovation Center for Soft Matter Science and Engineering, Beijing University of Chemical Technology, Beijing, 100029 China; 2 Hebei Chemical & Pharmaceutical College, Shijiazhuang, 050026 China; 3 Hebei Provincial Academy of Environmental Sciences, Shijiazhuang, 050037 China; 4 Bayin Guoleng Vocational and Technical College, Korla, 841002 China; 5 Key Laboratory of Industrial Ecology and Environmental Engineering (Ministry of Education), School of Environmental Science and Technology, Dalian University of Technology, Dalian, 116024 China; 6 Research Center for Eco-Environmental Sciences, Chinese Academy of Sciences, Beijing, 100085 China; 7 Hebei Provincial Meteorological Technical Equipment Center, Shijiazhuang, 050021 China

**Keywords:** Atmospheric chemistry, Atmospheric dynamics

## Abstract

Wintertime meteorological conditions are usually unfavorable for ozone (O_3_) formation due to weak solar irradiation and low temperature. Here, we observed a prominent wintertime O_3_ pollution event in Shijiazhuang (SJZ) during the Chinese New Year (CNY) in 2021. Meteorological results found that the sudden change in the air pressure field, leading to the wind changing from northwest before CNY to southwest during CNY, promotes the accumulation of air pollutants from southwest neighbor areas of SJZ and greatly inhibits the diffusion and dilution of local pollutants. The photochemical regime of O_3_ formation is limited by volatile organic compounds (VOCs), suggesting that VOCs play an important role in O_3_ formation. With the developed O_3_ formation path tracing (OFPT) approach for O_3_ source apportionment, it has been found that highly reactive species, such as ethene, propene, toluene, and xylene, are key contributors to O_3_ production, resulting in the mean O_3_ production rate (P_O3_) during CNY being 3.7 times higher than that before and after CNY. Industrial combustion has been identified as the largest source of the P_O3_ (2.6 ± 2.2 ppbv h^−1^), with the biggest increment (4.8 times) during CNY compared to the periods before and after CNY. Strict control measures in the industry should be implemented for O_3_ pollution control in SJZ. Our results also demonstrate that the OFPT approach, which accounts for the dynamic variations of atmospheric composition and meteorological conditions, is effective for O_3_ source apportionment and can also well capture the O_3_ production capacity of different sources compared with the maximum incremental reactivity (MIR) method.

## Introduction

Air pollution has caused widespread concern due to human health risks and economic losses^1,2^. Over the past few years, the Chinese government has made great efforts to improve air quality, especially by reducing fine particulate matter (PM_2.5_) concentrations. From 2013 to 2017, PM_2.5_ concentrations decreased by more than 30% across the country^3^. However, ozone (O_3_) concentrations show an increasing trend (around 2.9 ppbv yr^−1^) in many regions over the period 2013–2019^4^.

Tropospheric O_3_ is produced via the photochemical cycle of nitrogen monoxide (NO)-O_3_-nitrogen dioxide (NO_2_), which is sped up in the presence of volatile organic compounds (VOCs), resulting in an accelerated O_3_ production rate in the environment^5^. Xiong et al.^6^ investigated the VOCs pollution characteristics in Chengdu and found that combustion sources contributed more to VOCs in winter (23.9%) than that in summer (12.1%), and the main sources of VOCs were natural gas (NG)/liquefied petroleum gas (LPG) usage and industry. Seco et al.^7^ conducted seasonal (winter and summer) measurements at a forest site and found that biogenic emissions of isoprene and monoterpenes occurred only during the warmest summer months. Recent studies have found that wintertime VOC concentrations in Heibei province are 1.5 to 2 times higher than those in summer^8,9^. Therefore, the potential source of O_3_ in winter should be different from that in summer because O_3_ pollution in most Chinese cities is located in a VOC-limited regime and the emission sources and components of VOCs vary greatly^10,11^. In addition, photochemical reactions greatly depend on solar radiation and temperature^12^. Highly reactive VOCs (*k*_OH_ > 10^−11^ cm^3^ molecule^−1^ s^−1^, photochemical age assumed to be 12 h) may lose ~80% before being sampled due to photochemical loss in summer^13^. Previous studies investigated photochemical losses of VOCs in summer and found that total VOCs were underestimated by about 18.4–23.3% due to photochemical loss, while highly reactive species (isoprene, ethene, propene) were underestimated by about 30.0–61.9%^14,15^. Thus, the importance of these highly reactive VOCs to O_3_ formation might be overlooked in summer due to the photochemical losses based on observation-based modeling (OBM)^16,17^. The specific atmospheric conditions such as weak solar radiation and low temperature in winter should provide us a good opportunity to characterize the highly reactive VOCs, in particular, their roles in O_3_ production. However, most of the previous studies have mainly focused on summertime O_3_ pollution, while wintertime O_3_ pollution has attracted insufficient attention^12,18^.

Shijiazhuang (SJZ), the provincial capital of Hebei, is an important industrial base with 10 million residents. It is also an important site in the southwest transport channel of air pollutants in the North China Plain (NCP)^19^. Guan et al.^20^ investigated the characteristics and sources of summertime VOCs in Shijiazhuang using an offline sampling method. They found that oxygenated VOCs (OVOCs) accounted for 37.9% of the total VOCs, followed by alkanes (33.9%), unlike the results in Beijing^21,22^, Chengdu^6^, etc., where alkanes had the highest contribution (42.6–44.5%) to total VOCs. Meanwhile, petrochemical (24.2%) and other industrial sources (15.2%) were the main emission sources of VOCs in SJZ^20^. However, less attention has been paid to O_3_ pollution in SJZ at present although it is an urgent issue from the perspective of both regional transport of air pollutants and local photochemistry.

When we implement O_3_ pollution control, it is crucial to determine the main regional and sectoral sources of O_3_^23^. The trajectories analysis of air masses can identify the regional sources of surface O_3_, but not account for the chemical processes^24,25^, thus limiting its application in quantifying O_3_ sources. Emission-based model (EBM), which predicts the transport and photochemical reactions of O_3_ as well as its precursors utilizing air quality models with a known emissions inventory, is a powerful tool and has been widely used for O_3_ source apportionment^26,27^. The OBM, which simulates O_3_ formation processes constrained by measurements of the concentrations of O_3_, oxides of nitrogen (NO_x_), sulfur dioxide (SO_2_), carbon monoxide (CO), nitrous acid (HONO), VOCs, the photolysis rate constant (*J*), temperature (T), pressure (P), and relative humidity (RH), can avoid the uncertainties caused by emission inventories and the simulated dynamics of the boundary layer when compared with the EBM^28^. Thus, it has attracted much attention in China, in particular, due to the relatively insufficient research on emission inventories^25^. It has been well recognized that VOC species have different reactivities, resulting in different O_3_ production rates (P_O3_). However, previous studies have rarely quantified the contribution of different VOC sources to O_3_ production rates^29,30^. At present, a propylene-equivalent concentration method and a maximum incremental reactivity (MIR) method are usually used to calculate the reactivity of VOCs and the contributions of chemical species and sources to the ozone formation potential (OFP)^31,32^, because the production of O_3_ is VOCs-limited in the most of urban areas of China^33,34^. These two methods estimate the amount of O_3_ formed under optimum or ideal conditions, which may differ from the amount formed in the actual atmosphere. Ling et al.^35^ proposed an O_3_ source apportionment method combining positive matrix factorization (PMF) and OBM simulations, in which the OBM simulation was driven by the PMF extracted concentrations to calculate the relative O_3_ reduction efficiency (RORE) or relative incremental reactivity (RIR) of a single given VOC source. However, using a given VOC source as OBM input may result in differences in both the concentrations of free radicals and P_O3_ in the model from that in the actual situation. Therefore, there is a need to develop a more reliable method to apportion O_3_ sources and understand wintertime ozone pollution.

In this study, we propose an ozone formation path tracing (OFPT) approach for P_O3_ in the OBM under real observational conditions and combine it with the VOCs-PMF to quantify the sources of the O_3_ production rate. Based on an observation campaign in SJZ during the Chinese New Year (CNY) in 2021, we investigated the O_3_ pollution in SJZ from the perspective of both regional transport of air pollutants and local photochemistry. The contributions of anthropogenic VOCs, especially aromatics and alkenes with high reactivity, to O_3_ production and formation sensitivity regime were discussed. The OFPT method was used to identify the O_3_ sources from the perspective of O_3_ production. Meanwhile, the OFPT method was compared with the traditional one based on the MIR method. To the best knowledge, this is the first time report about the wintertime O_3_ pollution in SJZ and source apportionment in light of the P_O3_ based on the OFPT method. Our results highlight the role of highly reactive VOCs from industrial emissions in O_3_ pollution in SJZ.

## Results

### Air quality and meteorological conditions during the observation

**Fig. 1:**
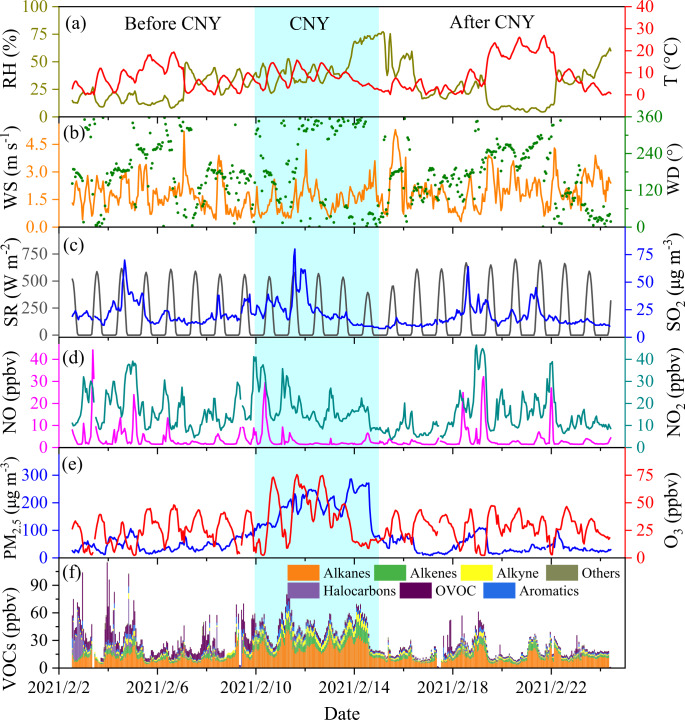
Changes in meteorological parameters and air pollutants during the observation period. Time series of meteorological parameters, including RH and T (**a**), WS and WD (**b**), and SR (**c**). Time series of air pollutants, including SO_2_ (**c**), NO and NO_2_ (**d**), PM_2.5_ and O_3_ (**e**), and VOCs (**f**). The shadow area represents the CNY period (10-14 February 2021).

Figure 1 shows the time series of meteorological parameters, including RH, T, solar radiation (SR), wind speed (WS), and wind direction (WD), and air pollutants (VOCs, NO_x_, O_3_, SO_2_, PM_2.5_) during our observations. The RH was 45.0 ± 10.8% during the Spring Festival period, which was higher than that before and after CNY (22.8 ± 14.7% and 28.0 ± 18.2%, respectively). The concentration of PM_2.5_ showed a similar trend to the RH, i.e., higher (187.3 ± 54.7 µg m^−3^) during CNY than that before and after CNY (46.7 ± 22.2 and 41.6 ± 25.3 µg m^−3^, respectively). The temperature ranged from 0 to 26.9 °C, with a mean value of 8.3 ± 6.3 °C during the observation period. The maximum daily 8-hour average (MDA8) O_3_ concentration increased significantly (*P* < 0.05) during CNY (48.8 ± 3.7 ppbv) when compared with that before (40.3 ± 3.2 ppbv) and after CNY (38.4 ± 2.7 ppbv), respectively. The maximal hourly O_3_ concentration even reached 75.5 ppbv during CNY, which is close to the Chinese second-order air quality standard (160 µg m^−3^, corresponding to 77.3 ppbv at 281 K and 1008 hPa). The WS was 1.5 ± 0.7 m s^−1^ during CNY, which was slightly lower than 1.7 ± 0.7 m s^−1^ before and 2.2 ± 0.9 m s^−1^ after CNY. Small wind speed is favorable for the accumulation of pollutants. Previous studies found that local emissions and chemistry significantly contribute to the accumulation of air pollutants when wind speeds were below 2 m s^−1^ ^36,37^. This implies that local emissions and chemistry may be the important contributors to O_3_ pollution during CNY. In addition, O_3_ pollution usually occurs in warm seasons under conditions of intense solar radiation and high ambient temperature^38^. The increased O_3_ concentration during CNY implies vigorous photochemistry although the meteorological conditions (e.g., lower T and SR) are not in favor of the occurrence of O_3_ pollution in winter.

As can be seen in Fig. 1f and Supplementary Table 1, the concentrations of alkanes, alkenes, alkyne, and aromatics increased significantly (*P* < 0.05) during CNY, with mean values of 23.3 ± 7.3, 7.3 ± 3.6, 5.2 ± 1.5, and 3.5 ± 1.7 ppbv, respectively, when compared with those before and after CNY period. The NO_x_ concentrations showed a slightly decreasing trend with the mean values of 21.7 ± 11.3, 20.9 ± 9.9, and 17.5 ± 12.3 ppbv before, during, and after CNY, respectively. The NO concentration was lower during CNY than that before and after CNY. This may be attributed to the enhanced generation rates of alkyl peroxide radicals (RO_2_) and hydrogen peroxide radicals (HO_2_) from VOCs during CNY, which subsequently will promote the conversion of NO to NO_2_. This is generally consistent with the slightly higher NO_2_ concentrations during CNY than that before and after CNY. It should be noted that the NO_x_ concentration is slightly higher during CNY compared to that (17.2 ± 7.3 ppbv) in the previous 4 days (Fig. 1e). This highlights the important role of VOCs in O_3_ pollution during CNY. As shown in Supplementary Fig. 1, CO concentrations increased significantly during CNY when compared with those before and after CNY, which implies the intensive combustion emissions from industry and/or residents in Shijiazhuang^39^.

### Regional transport *vs* local photochemistry

**Fig. 2:**
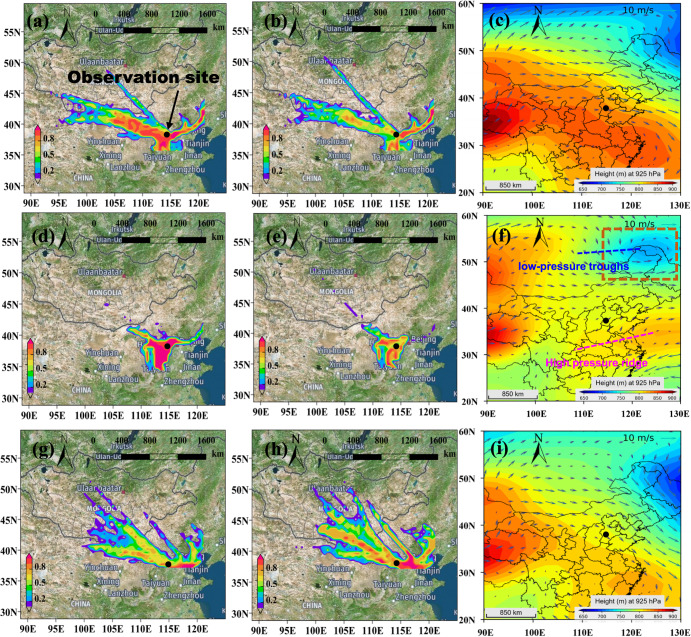
The potential source contribution function (PSCF) analysis of air pollutants and meteorological field changes during the observation period. The PSCF analysis of VOCs (**a**), O_3_ (**b**), and the field of geopotential height and wind (**c**) before CNY. **d**–**f** The PSCF analysis of VOCs (**d**), O_3_ (**e**), and the field of geopotential height and wind (**f**) during CNY, respectively. **g**–**i** The PSCF analysis of VOCs (**g**), O_3_ (**h**), and the field of geopotential height and wind (**i**) after CNY, respectively. The color bar in the left (**a**, **d**, **g**) and middle (**b**, **e**, **h**) columns indicates the possibility of the source, ranging from 0 to 1. The geopotential height in the right column (**c**, **f**, **i**) is at 925 hPa.

Although the mean wind speed on the surface was lower than 2 m s^−1^ during our observations, regional transportation of air pollutants (O_3_ and VOCs) might still be important. Figure 2 shows the potential source contribution of VOCs and O_3_ in different periods. VOCs and O_3_ have similar patterns of geographical sources regardless of the observation period although a slight difference is observable. If O_3_ mainly results from transport, it should show a different distribution pattern from that of VOCs, which are mainly from local emissions. Thus, the observed O_3_ pollution event at our observation site should be highly related to the photochemistry of VOCs besides the transport of O_3_^40^. Figure 2c, f and i show the fields of geopotential height and wind at a pressure of 925 hPa before, during, and after CNY, respectively. It can be seen that the pressure system in the NCP was influenced by the abnormal weakening of the West Pacific subtropical high-pressure system in the southern regions (Supplementary Fig. 2) and the temporary appearance of a low-pressure system in the northern regions of the observation site (dash box in Fig. 2f). Subsequently, the clean air masses from the northwest monsoon in the observation area are weakened, resulting in unfavorable dilution of local pollutants. Meanwhile, the area between the trough of low pressure and the ridge of high pressure is not conducive to local pollutants diffusion. This unfavorable meteorological condition can be further analyzed using the results of air mass trajectory.

Supplementary Fig. 3a, d and g show the 24-h backward trajectories of air mass at a height of 0.5 km before, during, and after CNY, respectively. The air mass was clustered to 3 trajectories in these three periods, according to the variation of the total variation of spatial (TVS) to a possible number of clusters (Supplementary Fig. 3b, e, h). Before and after CNY, long-distance trajectories from north-related directions (routes 1 and 1, 2, respectively) contributed 63.5–74.5% to the air masses (Supplementary Fig. 3a, g), while south and east directions (routes 2 and 3) with short-range trajectories dominated the air masses during CNY (69.1%, Supplementary Fig. 3d). As shown in Supplementary Fig. 3f, clusters 2 and 3 were near the ground surface with short-range trajectories during CNY. Meanwhile, cluster 1 and clusters 1&2 were transmitted at a high height during the periods before and after CNY, respectively. Long-range transport generally has higher wind speeds and a stronger dilution ability to air pollutants than short-range transport^41^. It should be noted that the southward ground surface winds only accounted for 24.0% during CNY (Fig. 1b), while the southward trajectories account for 45.8% during the same period (Supplementary Fig. 3d). This should be ascribed to their different heights, i.e., the start point of the air mass trajectory is 500 m from the ground (much less susceptible to topographic influence), while the wind direction is measured at 25 m at the observation station.

Interestingly, both the concentrations of O_3_ and VOCs well correlated with the relative contribution of short-range air masses (Supplementary Fig. 4). The highest contributions of the short-range air masses to VOCs and O_3_ were observed during CNY. In addition, high O_3_ concentrations generally come from the south direction, which coincided with the pollution distribution in SJZ (Supplementary Figs. 5 and 6). These results illustrate that the O_3_ pollution event during CNY should be mainly affected by the low-speed air mass intrusion from the southern regions of SJZ, leading to the accumulation of air pollutants. This can be ascribed to the fact that (1) the sudden changes in the meteorological system altered the pressure and wind fields at the observation site, which provides favorable conditions for the accumulation and reaction of pollutants during CNY; (2) the local primary emission sources of VOCs were located in the south of SJZ (Supplementary Fig. 6); (3) the urban areas of SJZ are semi-surrounded by the Taihang Mountains, and when east or south winds prevail (during CNY), local pollutants are blocked by the mountains, resulting in a constant accumulation of pollutants^42^; (4) the crowded industries with high emission rates of air pollutants in the neighboring areas^43^. Large-scale changes in meteorological conditions can affect local air quality. Therefore, unfavorable meteorological and topographic conditions lead to the occurrence of wintertime O_3_ pollution, which provides favorable conditions for the photochemistry of VOCs.

### Ozone formation sensitivity and contribution of reactive VOCs to O_3_ formation

**Fig. 3:**
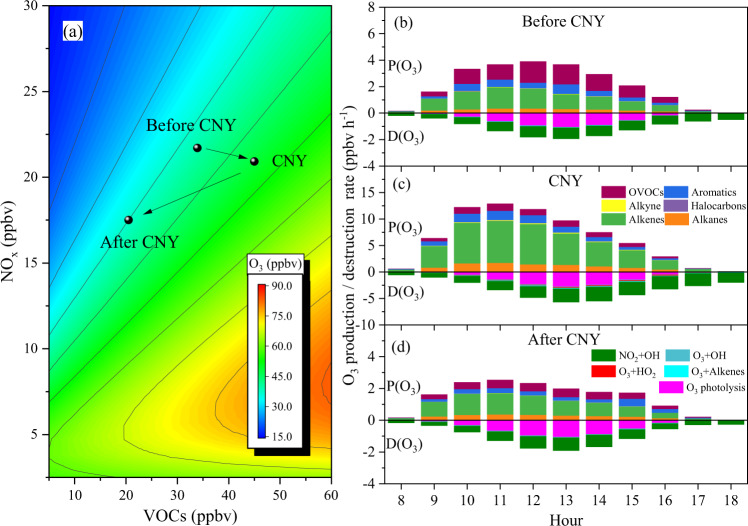
OBM simulation of O_3_. EKMA curves of MDA8 O_3_ concentration in winter in SJZ (**a**). **b**–**d** refer to the mean diurnal profile of O_3_ production rate (P_O3_) and destruction rate (D_O3_) before (**b**), during (**c**), and after CNY (**d**), respectively.

The empirical kinetic modeling approach (EKMA) curves are widely used to reveal the dependence of O_3_ formation on its precursors after the local pollution levels and meteorological parameters have been accounted for. As shown in Supplementary Fig. 7, the model well-predicted O_3_ concentrations with an *R*^2^ of 0.97 during the observations. Meanwhile, the simulated OH and HO_2_ concentrations are also at reasonable levels^44^. Figure 3a shows the O_3_ formation sensitivity during our observations. O_3_ formation was located in a VOC-limited regime as expected during our observations (Fig. 3a), while the ratio of VOCs/NO_x_ during CNY was significantly higher than those before and after CNY. During CNY overlapping the COVID-19 lockdown in 2020, it was also found that an emission reduction of NO_x_ led to an increase in O_3_ concentrations with a constant emission of VOCs in the North China Plain^18^. This means that O_3_ pollution during CNY in this study should be mainly ascribed to the enhanced VOC emissions. Furthermore, the emitted highly reactive species may also react with nitrate radicals (NO_3_) in the afternoon when NO concentrations are low, which reduces the atmospheric NO_x_ concentration and leads to a weak titration capability (Supplementary Fig. 8)^45^.

Figure 3b–d shows the mean diurnal variations of the P_O3_ and the D_O3_ during the three periods. The mean O_3_ production rates (P_O3_) were 2.2 ± 1.6, 6.4 ± 5.3, and 1.4 ± 1.2 ppbv h^−1^ before, during, and after CNY, respectively. The O_3_ production rates before and after CNY (Fig. 3b, d) were comparable with that in Beijing in winter (~3 ppbv h^−1^)^44^, while they were lower than that in Beijing in summer (10.7 ppbv h^−1^)^40^. During the observation, alkenes were the dominant contributors (53.5 ± 14.5%) to the P_O3_, followed by oxygenated VOCs (OVOCs, 20.1 ± 11.8%), aromatics (14.1 ± 4.7%) and alkanes (11.3 ± 3.7%). Similarly, alkenes accounted for 64.7 ± 8.7% of the total P_O3_ during CNY, followed by alkanes (12.5 ± 3.5%), aromatics (11.4 ± 2.6%), and OVOCs (10.1 ± 2.9%). The relative contribution of alkenes and aromatics to O_3_ formation increased around 4.9 and 3.1 times, respectively, during CNY when compared with before and after CNY periods. As shown in Supplementary Fig. 9, alkenes had the highest RIR value (0.43) during CNY, followed by aromatics (0.10), and OVOCs (0.08). A previous study found that OVOCs play an important role in RO_x_ (RO_x_ = OH + HO_2_ + RO_2_) formation (22–44%), subsequently, affecting the O_3_ production rate^46^. Thus, the RIR value of OVOCs was lower than that of alkenes and aromatics, but higher than that of alkanes (0.06) during CNY, as shown in Supplementary Fig. 9a. In terms of a single VOC compound, ethene, propene, toluene, and xylene showed high RIR values (Supplementary Fig. 9b–d). These four compounds accounted for 46.6 ± 7.1% of P_O3_ during CNY and 38.7 ± 10.1% of P_O3_ during the whole observation period. Thus, the O_3_ pollution event during CNY should be related to the increase in highly reactive VOCs. It should be noted that the peak values of P_O3_ usually appeared at around 11:00 a.m. This can be explained by the promotion effect of HONO on O_3_ formation because the photolysis of HONO is an important source of OH radicals in winter^47^. This is also supported by the maximal photolysis rate of HONO that appeared at 10:00 a.m. as shown in Supplementary Fig. 10.

The reaction between NO_2_ and OH was the major contributor to O_3_ destruction, which contributed 66.2% of the total D_O3_ during the whole observation period, followed by the photolysis of O_3_ (27.8%). These values are different from those summertime values reported in Beijing, Shanghai, Wuhan, Chengdu, and Lanzhou, e.g., photolysis of O_3_ and the reaction between NO_2_ and OH contributing 45.0–72.4% and 11.8–19.2% to the D_O3_, respectively^48^. This can be ascribed to different emission patterns of NO_x_ and solar irradiation in different seasons. These results also suggest that the reduction of NO_x_ concentrations during CNY (Fig. 1d) should also indirectly promote the accumulation of O_3_ by reducing the destruction of O_3_.

### Source apportionment of VOCs and O_3_ production

**Fig. 4:**
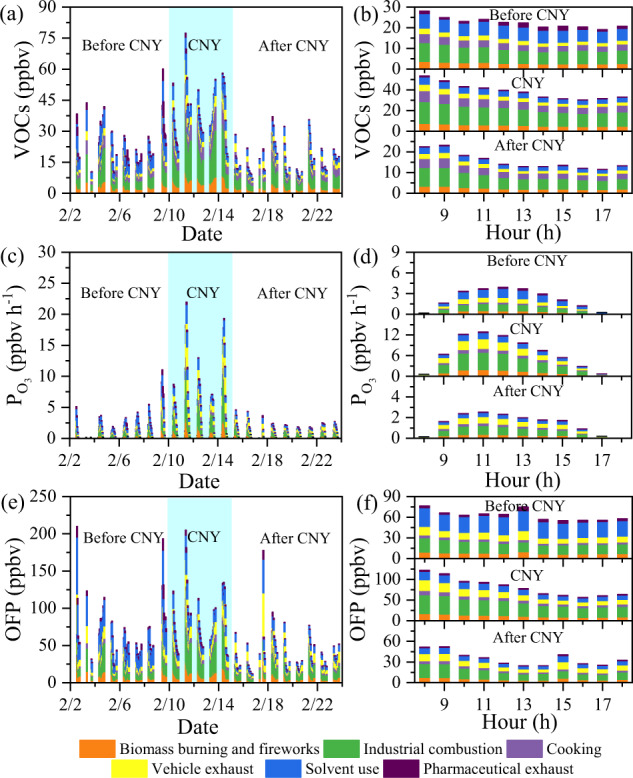
Comparison of the OFPT method with the OFP method for different sources of VOCs. The time series and diurnal variation of VOCs (**a**, **b**), P_O3_ (**c**, **d**), and OFP (**e**, **f**) during the daytime (08:00–18:00, local time).

To identify the sources of anthropogenic VOCs connecting with O_3_ pollution during CNY, source apportionment of the observed VOCs was performed using the PMF model. The source profiles are shown in Supplementary Fig. 11a, and the details about source identification are described in Supplementary Discussion 1. Briefly, six types of VOC sources were identified, including biomass burning mixed with fireworks, industrial combustion, cooking, vehicle exhaust, solvent use, and pharmaceutical process exhaust. Figure 4a shows the time series of each source’s contribution to VOCs. As shown in Supplementary Table 2 and Supplementary Fig. 11, the order of importance for the sources was industrial combustion (29.4–41.2%), solvent use (13.8–25.4%), biomass burning/fireworks (14.2–16.7%), cooking (13.3–19.5%), vehicle exhaust (8.8–10.8%), and pharmaceutical exhaust (1.7–6.3%).

For comparison, summertime VOC observations were also performed at the same observation site from June 24 to July 27, 2021 (Supplementary Fig. 12). Besides these six sources of VOCs in winter, a biogenic source characterized by high loading of isoprene was also identified in summer. The relative contributions of these sources in summer are in the following order, vehicle exhaust (26.2%) > industrial combustion (24.0%) > cooking (21.7%) > solvent use (9.2%) > biomass burning (9.0%) > biogenic emissions (8.1%) > pharmaceutical exhaust (1.2%). Thus, the contribution of industrial combustion to the observed VOCs (37.8%, 8.9 ± 5.6 ppbv) was higher in winter than that in the summer, i.e., 24.0% (Supplementary Fig. 12c, 2.3 ± 0.9 ppbv). In addition, it can be seen from Supplementary Table 1 that the contributions of OVOCs and aromatics to the total VOCs in summer (25.6% and 11.1%, respectively) are higher than those in winter (17.9% and 7.3%, respectively), while the contributions of alkenes and alkyne to the total VOCs are lower in summer (6.3% and 4.4%, respectively) than those in winter (13.9% and 9.1%, respectively). These results indicate that the role of highly reactive species such as alkenes and alkyne in O_3_ formation is visible due to the weak irradiation in winter, while it might be underestimated in summer.

To assess the effect of different sources on O_3_ production capacity, we calculated P_O3_ and OFP using the OFPT method and MIR method, respectively. The latest MIR values are used to calculate the OFP^49^. Figure 4e, f shows the contribution of each VOC source to the O_3_ production rate according to the OFPT method. The largest source of the O_3_ production rate was industrial combustion (2.6 ± 2.2 ppbv h^−1^, 39.9 ± 3.7%) during CNY, followed by vehicle exhaust (1.3 ± 1.3 ppbv h^−1^, 20.6 ± 3.6%), biomass burning/fireworks (0.9 ± 0.8 ppbv h^−1^, 14.7 ± 0.6%), solvent use (0.9 ± 0.8 ppbv h^−1^, 14.3 ± 1.0%), cooking (0.6 ± 0.5 ppbv h^−1^, 8.6 ± 1.6%), and pharmaceutical exhaust (0.1 ± 0.1 ppbv h^−1^, 1.9 ± 1.0%). For the MIR method, as shown in Fig. 4e and Supplementary Fig. 11c, industrial combustion contributed to 33.0 ± 14.3 ppbv (40.5 ± 3.7%) of the OFP during CNY, and the second important source contributing to the OFP was vehicle exhaust (14.5 ± 8.8 ppbv, 17.3 ± 2.9%), followed by solvent use (13.6 ± 5.5 ppbv, 17.1 ± 1.1%), biomass burning/fireworks (11.2 ± 5.1 ppbv, 13.7 ± 0.5%), cooking (7.0 ± 3.0 ppbv, 8.8 ± 1.3%), and pharmaceutical exhaust (2.1 ± 1.2 ppbv, 2.6 ± 1.0%). As shown in Fig. 4b, f, VOCs and OFPs had similar daily variation patterns, e.g., low values presenting at noon, because the OFPs were obtained by multiplying the VOC concentration and the MIR coefficient, which is a constant value under the specific condition being most sensitive to VOCs^49–51^. Thus, the OFP calculated with the MIR coefficient cannot reflect the actual atmospheric O_3_ production in terms of diurnal variations, although the order of the source contribution to O_3_ formation is similar to that of P_O3_ (Supplementary Figs. 11c, d or Supplementary Table 2). Meanwhile, the P_O3_ showed a similar diurnal pattern with O_3_ concentration in the daytime, but different from that of VOCs or OFP, as shown in Fig. 4d. The source apportionment based on the O_3_ production rate should be more accurate than that based on the MIR method due to the following reasons. (1) The O_3_ production rates are calculated based on the observed chemicals and meteorological parameters with a high-time resolution rather than that at fixed values of MIR because the formation of O_3_ is highly correlated with free radicals in the daytime^44,52,53^. (2) The mean change rate of measured O_3_ (dc_O3, Measured_/dt) and net P_O3_ (have considered the O_3_ loss) are comparable during the observation period with the peak values of 4.3 and 5.1 ppbv h^−1^ at 10 a.m., respectively, and the OFP is obviously different from the actual situation (Supplementary Fig. 13). (3) The P_O3_ is better to indicate the O_3_ pollution than the OFP during the whole observation period. For example, similar to the O_3_ concentrations, high P_O3_ values only occurred during CNY, while high OFP values appeared in all three periods (Figs. 1 and 4). (4) The P_O3_ generally shows similar diurnal variation patterns with O_3_ during the observation period, while the OFP shows opposite variation patterns with O_3_ (Fig. 4 and Supplementary Fig. 8). Therefore, the P_O3_ is able to correctly reflect the actual diurnal variations of each source to O_3_ formation.

It should be noted that the total P_O3_ during CNY was 3.7 times higher than before and after CNY. Besides being the largest source of the P_O3_ (2.6 ± 2.2 ppbv h^−1^) during CNY, industrial combustion showed the biggest increment (4.8 times) regarding the P_O3_ during CNY when compared with the mean value before and after CNY. This is consistent with the fact that the air masses during CNY were dominantly from the southern and eastern regions in the local and neighboring areas (Fig. 2), featured by intensive industrial emissions. In addition, the RIR values of high reactive species (ethene, propene, toluene, benzene, and xylene) from industrial combustion accounted for 36.5% of the total RIR value during CNY (Supplementary Fig. 14). These results highlight the importance of industrial emissions in O_3_ formation in Shijiazhuang, and highly reactive species play an important role under the conditions of weak irradiation and low temperature in winter. The different emission reduction scenarios of industrial sources were also further studied as shown in Supplementary Fig. 15. It can be seen that an 80-100% reduction of industrial emissions during CNY is needed to return to the O_3_ levels before CNY, which highlights the importance of industry in the observed O_3_ pollution event. Meanwhile, the P_O3_ attributed to vehicle exhaust and biomass burning/fireworks with large absolute values (1.3 and 0.9 ppbv h^−1^) also showed obvious increases (3.7–3.8 times) during CNY. Thus, attention should also be paid to these local sources of O_3_ pollution.

Although the VOC concentrations in summer (Supplementary Fig. 12d) were lower than that in winter, the O_3_ production rates in summer (13.1 ± 10.0 ppbv h^−1^) were larger than that in winter (2.9 ± 3.8 ppbv h^−1^) due to the stronger solar radiation. Meanwhile, the emission sources of VOCs varied obviously between winter and summer. Thus, the dominant source of O_3_ also changed obviously. For example, the summertime sources of O_3_ follows the order of: vehicle exhaust (3.3 ± 2.7 ppbv h^−1^, 25.0 ± 7.7%) > biogenic (2.7 ± 2.7 ppbv h^−1^, 20.4 ± 18.6%) > industrial combustion (2.4 ± 1.9 ppbv h^−1^, 18.4 ± 3.7%) > cooking (1.6 ± 1.3 ppbv h^−1^, 11.9 ± 3.6%) > solvent use (1.5 ± 1.4 ppbv h^−1^, 11.8 ± 8.2%) > biomass burning (1.3 ± 1.1 ppbv h^−1^, 10.3 ± 3.6%) > pharmaceutical exhaust (0.3 ± 0.3 ppbv h^−1^, 2.3 ± 1.3%). In particular, biogenic VOCs only contributed 8.1% to the total VOCs in summer, while it was the second largest source of the P_O3_. Industrial combustion, as the observed largest O_3_ source in winter, became the third source of O_3_ in summer. In addition, to understand the influence of the components of VOCs on O_3_ production, a cross-simulation of meteorological conditions was performed. As shown in Supplementary Table 3, the P_O3_ of industrial combustion increased from 2.6 to 4.1 ppbv h^−1^ when using summer meteorological conditions during CNY. The virtual P_O3_ (4.1 ± 3.6 ppbv h^−1^) is even higher than the P_O3_ in summer (2.4 ± 1.9 ppbv h^−1^). The relative contribution of alkenes to P_O3_ in winter (52.8%) was also higher than that in summer (43.1%) as shown in Supplementary Fig. 16. These results highlight the highly reactive VOCs from industrial combustion in wintertime O_3_ photochemistry, while they might be overlooked in summer due to chemical loss.

## Discussion

Although the solar radiation was much weaker in wintertime compared to that in summer, wintertime O_3_ pollution events still occurred in SJZ during our observations. The weak solar radiation facilitates the measurement of highly reactive VOCs. The high RIRs and concentrations of alkenes highlight their importance to O_3_ formation in winter. In summer, the contribution of these highly reactive species might be underestimated due to photochemical loss. The potential source contribution function (PSCF) results showed the vital role of photochemistry in the observed O_3_ pollution event. What’s more, O_3_ pollution during CNY was strongly affected by the changes in weather systems, e.g., the abnormal weakening of the West Pacific subtropical high-pressure system in the southern regions and the temporary appearance of the cut-off low-pressure system in the northern regions of the observation site, which provided favorable conditions for the accumulation and reaction of pollution during CNY. Further analysis in combination with the trajectories of air masses reveals that the levels of VOCs and O_3_ were well correlated with the ratio of short-ranged trajectories from the southern and eastern regions, implying that local and neighboring emissions contributed to the occurrence of O_3_ pollution events during CNY. Highly reactive species such as ethylene, propene, toluene, and xylene with high RIR values positively correlated with O_3_ production during CNY.

To accurately quantify the contribution of different VOC species to ozone production, the OPFT method was developed by tracing the O_3_ production rate in different generations of VOC oxidation in the OBM, which paves the way for source apportionment of the O_3_ production rate. The OPFT method reflects the dynamic O_3_ production under atmospheric conditions at an observational site unlike the O_3_ source apportionment method based on the MIR values at a fixed atmospheric scenario. In addition, the OPFT method does not interrupt the ratio of VOCs to NO_x_, which is a problem for the O_3_ production rate source apportionment based on the RORE or RIR method^35^, during box model simulations. Thus, the OPFT provides more refined and accurate information on O_3_ sources when compared with these previous studies. Our results also confirmed that the sequence of O_3_ source contribution based on the OFPT method is credible when compared with that based on the MIR method if O_3_ production is in a VOC-limited regime. Moreover, the source apportionment of the P_O3_ based on the OFPT method could capture not only the diurnal variations of O_3_ formation but also the long-term time series of O_3_ pollution in the ambient environment.

With the aid of the developed OFPT approach, the wintertime O_3_ pollution event was analyzed in detail. The OFPT results found that the increased O_3_ concentrations during CNY could be ascribed to enhanced O_3_ production related to industrial combustion, vehicle exhaust, and biomass burning/fireworks, from which highly reactive VOCs were emitted during the CNY. A comparison with the summer results further confirms that highly reactive VOCs were visible in winter due to the lower radiation. It should be pointed out that the OFPT method in this study does not account for vertical or horizontal transport because it is based on a box model. A combination with a 3D air quality model should provide a more comprehensive understanding of O_3_ pollution in the future.

## Methods

### Field measurements

The sampling site (Hebei Atmospheric Super Station, 38.03°N, 114.61°N) is located on the rooftop of the main building of Shijiazhuang University, around 25 meters from the ground and around 250 m from Zhujiang Road in SJZ. The observations were carried out from February 2 to 24, 2021, covering before (February 2, 2021, to February 9, 2021), during (February 10, 2021, to February 14, 2021), after CNY (February 15, 2021, to February 24, 2021), and from June 24 to July 27, 2021. The instruments used in this study are summarized in Supplementary Table 4. Briefly, the concentrations of VOCs (82 species) were measured with a Gas Chromatography-Mass Spectrometry/Flame Ionization Detection (GC-MS/FID, EXPEC2000-MS). Trace gases, including NO_x_, SO_2_, CO, and O_3_, were measured with the corresponding analyzer (Thermo Scientific, 42i, 43i, 48i, and 49i). HONO was measured with a Monitoring AeRrosols and Gases in Ambient Air (MARGA, ADI 2080). PM_2.5_ was measured with a Beta Attenuation Mass Monitor (BAM-1020, Met One Instruments). Meanwhile, meteorological conditions, including temperature, pressure, relative humidity, wind speed, and direction, were monitored using a weather station (WXT 520, Vaisala). More information about the instruments and data quality can be seen in Supplementary Note 1.

### Ozone formation path tracing approach (OFPT)

O_3_ formation was calculated using the Master Chemical Mechanism (MCM, v3.3.1, http://mcm.leeds.ac.uk/MCM/). Simulations were performed with the Framework for 0-D version Atmosphere Modeling (F0AM) software package^54,55^, and constrained by meteorological parameters (T, RH, P), air pollutants (VOCs, NO_x_, CO, CH_4_, HONO, SO_2_), photolysis frequencies (*J* values). The *J* values were calculated by the National Center for Atmospheric Research tropospheric ultraviolet and visible (TUV) transfer model (http://www.acd.ucar.edu/TUV), which considers the influence of aerosols and O_3_, and further corrected according to Eq. 1 to account for the effect of clouds.

1 $${{J}}_{{\rm{corrected}}}\,=\,{{J}}_{{\rm{TUV}}}\,\times \,\frac{{{\rm{SR}}}_{{\rm{cloud}}}}{{{\rm{SR}}}_{{\rm{clear}}}}$$

Where *J*_corrected_ and *J*_TUV_ represent the corrected and calculated values of photolysis rate, respectively; SR_cloud_ and SR_clear_ represent the downward solar radiation under cloudy and clear conditions, respectively.

Seventy-two VOC species were selected as OBM inputs (Supplementary Table 5), which accounted for 96.3 ± 2.5% of the total VOC concentrations during the observation period. The P_O3_ attributed to each species was extracted during OBM simulations using the model’s built-in ExtractRates function. Each step of the simulation is set to link. The P_O3_ was calculated according to Eq. 2, in terms of the oxidation rate of NO to NO_2_ by peroxyl radicals (RO_2_ and HO_2_). The branching reaction to form RONO_2_ does not affect the P_O3_ calculations because it is not traced in the OBM. The destruction rate of O_3_ (D_O3_) was calculated according to Eq. 3, in which photolysis of O_3_ (represented by O^1^D loss to H_2_O) and the reactions of O_3_ with OH, HO_2_, and alkenes are accounted for^40,56^. In addition, the reaction between NO_2_ and OH leads to a net loss of O_3_ in the daytime, thus it is also considered in Eq. 3^56^.

2 $${{\rm{P}}}_{{{\rm{O}}}_{3}}{={\rm{k}}}_{{{\rm{HO}}}_{2}+{\rm{NO}}}[{\rm{HO}}_{2}][{\rm{NO}}]{+{\rm{k}}}_{{{\rm{RO}}}_{2}+{\rm{NO}}}[{{\rm{RO}}}_{2}][{\rm{NO}}]$$

3 $$\begin{array}{c}{{\rm{D}}}_{{{\rm{O}}}_{3}}{={\rm{k}}}_{{{\rm{O}}}^{1}{{\rm{D}}+{\rm{H}}}_{2}{\rm{O}}}[{{\rm{O}}}^{1}{{\rm{D}}}][{\rm{H}}_{2}{\rm{O}}]+\left(\right.{\rm{k}}_{{{\rm{O}}}_{3}+{\rm{OH}}}[{\rm{OH}}]+{\rm{k}}_{{{\rm{O}}}_{3}+{{\rm{HO}}}_{2}}[{{\rm{HO}}}_{2}]\\+\,{\rm{k}}_{{{\rm{O}}}_{3}+{\rm{Alkenes}}}[{\rm{Alkenes}}]\left)\right.[{{\rm{O}}}_{3}] +{\rm{k}}_{{{\rm{OH}}+{\rm{NO}}}_{2}}[{\rm{OH}}][{{\rm{NO}}}_{2}]\end{array}$$

where *k*_i_ means the corresponding reaction rate; [*i*] is the concentration of the species *i*.

**Fig. 5:**
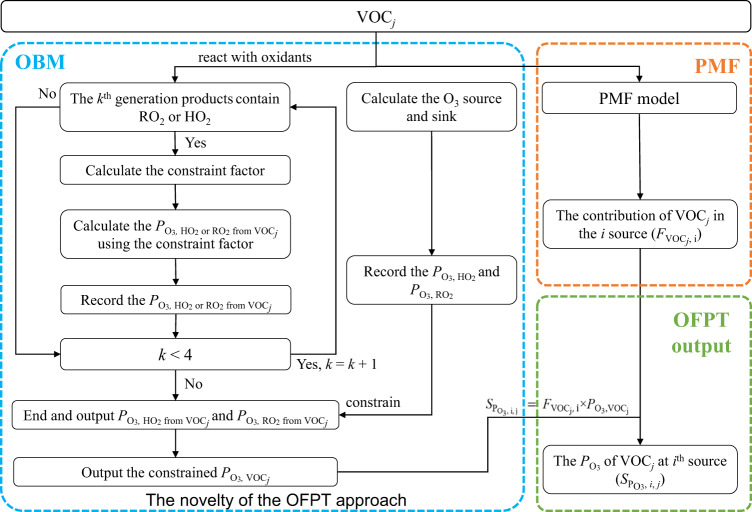
The workflow of the OFPT method. The flow of the OFPT approach explains the calculation process of the contribution of different VOCs to O_3_ production. The steps in the blue box are calculated using OBM, the steps in the orange box are calculated using PMF, and the step in the green box is calculated using Eq. 4.

Figure 5 shows the workflow of the OFPT method to obtain the P_O3_ of each VOC. Briefly, for a VOC_*j*_, RO_2_, and HO_2_ are searched in all the first-generation products after the reactions are initialized by oxidants (OH, NO_3_, and O_3_). If RO_2_ or HO_2_ is found, the corresponding reaction rates of HO_2_/RO_2_ with NO are recorded. Subsequently, all the first-generation products will further react with oxidants. RO_2_ and HO_2_ will be searched in all second-generation products again. If RO_2_ or HO_2_ is present, a constraint factor needs to be determined by performing a source-sink analysis of its precursors. The cause is that the traced RO_2_ or HO_2_ can be generated from both the first-generation products of the VOC_*j*_ and other VOCs as well as their products. Then, the factor is used to constrain the reaction rate of the searched RO_2_ or HO_2_ with NO. The third-generation reactions are treated in the same way. The reaction rates for the fourth-generation products are close to zero, thus we only account for three generations of RO_2_ or HO_2_. In addition, considering that not all RO_2_ and HO_2_ radicals eventually generate O_3_ due to homogeneous or non-homogeneous losses^57^, the P_O3, HO2_ and P_O3, RO2_ obtained from the source-sink equilibrium of O_3_ are used to constrain the P_O3, HO2_ and P_O3, RO2_ from the VOC_*j*_, respectively. Moreover, besides oxidants, photolysis is also considered in the OFTP method.

VOC source apportionments were calculated by the PMF model (US EPA 5.0). In this study, forty-two species were selected for PMF analysis according to the principles as shown in Supplementary Note 2. The selected VOCs accounted for 94.3 ± 6.9% of the total observed VOC concentrations with an *R*^2^ of 0.7, and 82.7 ± 11.9% of the total P_O3_ during the observation period. More information about PMF model setup and validation can be seen in Supplementary Note 2. A six-factor solution for wintertime VOCs was identified in this study. The contribution of each VOC source to the P_O3_ at a given time was further calculated by multiplying the fraction of the VOC attributed to a source and its corresponding P_O3_ traced in the OBM, i.e.,

4 $${{\rm{S}}}_{{{\rm{P}}}_{{{\rm{O}}}_{3},{\rm{i}}}}=\mathop{\sum }\limits_{{\rm{j}}=1}^{{\rm{n}}}({{\rm{F}}}_{{{\rm{VOC}}}_{{\rm{j}}},{\rm{i}}}{\times {\rm{P}}}_{{{\rm{O}}}_{3}{,{\rm{VOC}}}_{{\rm{j}}}})$$

where *S*_PO3,i_ is the source contribution of the *i*th VOC source including *n* VOC species to O_3_ production rate at a given time (ppbv h^−1^); *F*_VOCj,i_ is the relative contribution of a VOC_*j*_ to the *i*th VOC source (%), and *P*_O3, VOCj_ is the O_3_ production rate of a VOC_*j*_ at a given time (ppbv h^−1^) according to the corresponding product rate of RO_2_ and HO_2_ produced from the VOC_*j*_.

### Empirical kinetic modeling approach and RIR calculation

The EKMA is used to describe the nonlinear relationship between O_3_ and its precursors NO_x_ and VOCs^44^. To assess the O_3_ formation regime, we adopted the OBM to simulate O_3_ concentration isodepth by varying VOCs and NO_x_ concentrations from 10% to 200% of their mean values during the observation period, while taking the mean values of all other inputs. O_3_ isopleths were plotted using the averaged MDA8 O_3_ concentrations. According to a previous study, O_3_ formation is promoted by HONO, which is an important source of OH radicals^58^. Thus, HONO was accounted for in the model simulations in our study.

To identify highly reactive species, the RIR method was used to evaluate the sensitivity of different precursors to O_3_ production^59^. Briefly, we changed the model input for a target precursor to a certain extent and obtained the correspondingly relative changes of P_O3_ compared to that of the target species, i.e.,

5 $${\rm{RIR}}({\rm{X}})=\frac{\frac{{\Delta {\rm{P}}}_{{{\rm{O}}}_{3}}({\rm{X}})}{{{\rm{P}}}_{{{\rm{O}}}_{3}}({\rm{X}})}}{\frac{\Delta {\rm{c}}({\rm{X}})}{{\rm{c}}({\rm{X}})}}$$

where *c*(X) and Δ*c*(X) are the measured concentration and the concentration changes of a precursor *X* (ppbv), respectively; P_O3_ and ΔP_O3_ represent the simulated P_O3_ in the base scenario and the changes of P_O3_ resulting from the concentration changes of the precursor. In this study, a relative change of 20% was used as the same as that in a previous study^52^. The O_3_ concentrations were calculated in the daytime (8:00–18:00 local time).

### Potential source contribution function analysis

The PSCF analysis was performed to understand the possible spatial distribution of the emission sources, by which the air mass transport trajectories at the observed site are calculated^60^. The 24 h backward trajectories of air mass were calculated with a 1-h temporal resolution from a global database using MeteoInfo software^61^. The area was divided into 0.5° × 0.5° grid to study VOCs and O_3_ potential sources. Furthermore, a cluster analysis method was also performed to study the variation of the air mass trajectories during the observation period.

## Supplementary information

Supplementary Information for The contribution of industrial emissions to ozone pollution: Identified using ozone formation path tracing approach

## Data Availability

The data of air mass backward trajectories are available at ftp://arlftp.arlhq.noaa.gov/. The data of geopotential height and the wind is available at http://www.cpc.ncep.noaa.gov/. Solar radiation (SR) was obtained from Copernicus Services (www.copernicus.eu/en). All additional data to perform the analyses are available upon reasonable request from the corresponding author (liuyc@buct.edu.cn).

## Code availability

All additional codes needed to perform the analyses are available upon reasonable request from the corresponding author (liuyc@buct.edu.cn).
