# Supplementary material for: The contribution of industrial emissions to ozone pollution: identified using ozone formation path tracing approach
Source: NPJ Clim Atmos Sci. 2023 May 16;6(1):37. doi: 10.1038/s41612-023-00366-7 (PMC10186276; doi:10.1038/s41612-023-00366-7)
Supplement: Supplementary file 1 — Supplementary Information for The contribution of industrial emissions to ozone pollution: Identified using ozone formation path tracing approach [file 41612_2023_366_MOESM1_ESM.pdf]

Supplementary Information for

The contribution of industrial emissions to ozone pollution: Identified using ozone formation path tracing approach

Junlei Zhan, Wei Ma, Boying Song, Zongcheng Wang, Xiaolei Bao*, Hong-Bin Xie, Biwu Chu, Hong He, Tao Jiang, Yongchun Liu*

*Correspondence: liuyc@buct.edu.cn or bxl5@163.com

Supplementary Information consists of 1 discussion, 2 notes, 18 figures and 6 tables.

Supplementary Discussion 1

Source identification

Factor 1 exhibits high mixing ratios of methylchloride (1.02 ppbv, 100%), ethene (0.59 ppbv, 16.2%), acetylene (0.40 ppbv, 14.9%) and aromatics such as toluene (0.12 ppbv, 14.8%) and benzene (0.12 ppbv, 12.7%). The methylchloride is considered to be a marker for biomass burning [1]. In addition, previous studies have considered potassium (K) as a tracer of biomass burning as well, especially in winter when biomass combustion activity is high [2,3]. Meanwhile, K and strontium (Sr) were also considered as tracers of local firework [4,5]. The correlation coefficient (r) between Factor 1 and K is 0.7, and the correlation coefficient between Factor 1 and Sr is 0.6, as shown in Supplementary Fig. 17A. The emissions from biomass burning and fireworks were not significantly increased during CNY in the Shijiazhuang area due to the travel restrictions and the ban on fireworks displays in urban areas.

Factor 2 has high loadings of ethene (2.86 ppbv, 41.4%), ethane (2.28 ppbv, 62.3%), acetylene (1.67 ppbv, 61.6%), benzene (0.67 ppbv, 72.3%) and toluene (0.25 ppbv, 30.6%). Acetylene is considered to be a combustion indicator [6]. Meanwhile, Shijiazhuang is a typical industrial city, and the emission distribution and capacity are shown in Supplementary Fig. 6. The ratio of benzene/toluene was 2.68, which was close to the industrial combustion (2.62) reported by Chen et al. [7] Furthermore, carbon disulfide (CS_2) is considered the industrial process product, such as the Claus gas desulfurizing process [8].

Factor 3 mainly consists of abundant propane (1.22 ppbv, 35.0%), isobutane (0.32 ppbv, 45.1%) and n-butane (0.51 pbbv, 47.7%). The i-butane/n-butane ratio of approximately 0.55 is close to the characteristic value of LPG/NG, which is attributed to cooking [9]. In addition, high concentration of i-pentane (0.23 ppbv, 33.5%), n-pentane (0.18 ppbv, 43.8%) methylcyclopentane (0.02 ppbv, 62.0%), and n-hexane (0.06 ppbv, 43.7%) were also found in Factor 3. Cheng et al. [10] investigated the cooking profile and found that cooking releases a large number of alkanes such as i-butane, i-pentane, n-

pentane, etc., which is generally consistent with the Factor 3 profile.

Factor 4 is characterized by the high contents of propene (0.18 ppbv, 44.6%), cis/trans-2-butene (0.01 ppbv, 59.7%; 0.01 ppbv, 51.9%), i/n-pentane (0.26 ppbv, 36.8%; 0.13 ppbv, 31.4%), methyl tert-butyl ether (MTBE) (0.05 ppbv, 63.1%) and aromatics, such as m/p-xylene (0.09 ppbv, 44.6%) and o-xylene (0.03 ppbv, 40.5%). The n-pentane and i-pentane are typical tracers of volatile gasoline [11,12]. The percentage ratio of n/i-pentane was 1.2, which is close to the 1.1 reported by Liu et al. Meanwhile, MTBE is usually used as a gasoline additive to improve octane ratings [1,13]. In addition, it also found that Factor 4 has a well correlation with NOx as shown in Supplementary Fig. 17B, which has been considered as vehicle emissions [14].

Factor 5 contains high levels of OVOCs, including acetaldehyde (1.29 ppbv, 84.2%), acetone (0.74 ppbv, 60.3%), ethyl acetate (0.15 ppbv, 15.9%), and aromatics, which used as solvents in chemical manufacturing processes. In addition, dichloromethane (0.04 ppbv, 43.2%) and freon-11 (0.05 ppbv, 36.1%) were also found in Factor 5. OVOCs (acetaldehyde, acetone, ethyl acetate) and halocarbon (dichloromethane) are used as industrial solvents and adhesives in the automotive coating, and furniture manufacturing [1,15,16]. Aromatics are the major component of paint [17]. Meanwhile, freon is also applied to synthetic resin manufacturing as a blowing agent.

Factor 6 has large amounts of ethyl acetate, tetrahydrofuran, and butanal, which are identified as emissions from pharmaceutical processes [18,19].

Supplementary Note 1

Sampling instrument and data quality control

All instruments are located in an air-conditioned room and the sample tubes are wrapped with heating jackets and insulation to ensure that the temperature remains stable between 22 and 27°C. NOx, SO_2, CO, and O_3 analysers (42i, 43i, 48i, 49i, respectively, Thermo-Fisher Scientific, USA) were used to measure the concentrations of the corresponding gas species. HONO was detected by an analyzer (Marga- ADI 2080, Applikon Analytical B.V., the Netherlands) with 1h of time resolution. The $PM_{2.5}$ concentration was measured by a beta attenuation mass monitor (BAM-1020, Met One Instruments Inc., USA) coupled with a smart heater (BX-830, Met One Instruments Inc., USA) to control the RH (around 35%) and a $PM_{2.5}$ inlet (URG) for cut off the particle's diameter larger than 2.5μm. VOCs were measured by an online GC-MS/FID instrument (EXPEC2000-MS, Expec-Tech., China), which consisted of two independent low carbon (C_2-C_5) and high carbon (C_6-C_{12}) analyzers. The low carbon (C_2-C_5) and high carbon (C_6-C_{12}) components were detected by flame ionization detector (FID) and mass spectrometer (MS) detector, respectively. External calibrations were performed monthly, using standard gas mixtures of volatile organic compounds (PAMS and TO-14, Linde Gas, USA). Six gradient concentrations were set for each calibration and each concentration was repeated three times. In this study, a total of 82 VOCs (including 21 alkanes, 1 alkyne, 11 alkenes, 18 OVOCs, 14 aromatics, 16 halo hydrocarbons, and 1 other) were analyzed with a limit of quantification of 0.01-0.1 ppbv as shown in Supplementary Table 5. The relative standard derivations (RSDs) of each VOC species were within 7% for each compound among seven repeated experiments.

Supplementary Note 2

PMF model setup and validation

Positive matrix factorization (PMF) analysis was carried out to identify the potential VOC emission sources. PMF is an effective tool for multi-factor analysis to determine the number of potential sources of pollutants and the corresponding source contribution, which have been described in detail [20,21]. The concentrations of VOCs during our observations were input into the PMF model for VOC source apportionment. The species with S/N > 1 were categorized as strong; those with 0.5 < S/N < 1 were categorized as weak; those remaining were categorized as bad. Meanwhile, species missing >25% of samples or having a high proportion (>35%) of samples below the method detection limit (MDL) were excluded [22]. The uncertainty is calculated by Supplementary Equation 1 when the measured concentration is greater than MDL, and the rest by Supplementary Equation 2.

$$\mathrm{Unc} = \sqrt{(\mathrm{Error\ Fraction} \times \mathrm{Concentration})^2 + (0.5 \times \mathrm{MDL})^2} \quad (1)$$

$$\mathrm{Unc} = \frac{5}{6} \times \mathrm{MDL} \quad (2)$$

We have tested the $Q_{expected}/Q_{robust}$ value as shown in Supplementary Fig. 18. We found that when the $Q_{expected}/Q_{robust}$ value gradually decreased as the number of factors increased from 3 to 9, the $Q_{expected}/Q_{robust}$ value flattens out when the factor number is 6, at which point the $Q_{expected}/Q_{robust}$ value (1.29) is close to one. Theoretically, for the optimal solution, the rate of change of $Q_{expected}/Q_{robust}$ value is the most stable [20,23]. Accordingly, 6 major pollution sources were identified. After identifying the six-factor solution, the disproportionate effect of the panel observations on the solution was detected and estimated using the bootstrap (BS) method, which estimates the effect of rotational ambiguity to some extent. The BS dataset is constructed by randomly sampling the observations from the original dataset. The base run with minimum $Q_{expected}/Q_{robust}$ value was provided to map each BS run. The average R^2 value of VOC species was 0.7. After 100 BS bootstrap runs, the mapping results of six bootstrap

factors to base factors were shown in Supplementary Table 6. Mapping over 80% of the factors indicates that BS uncertainties can be interpreted and that the number of factors may be appropriate.

Supplementary Figures

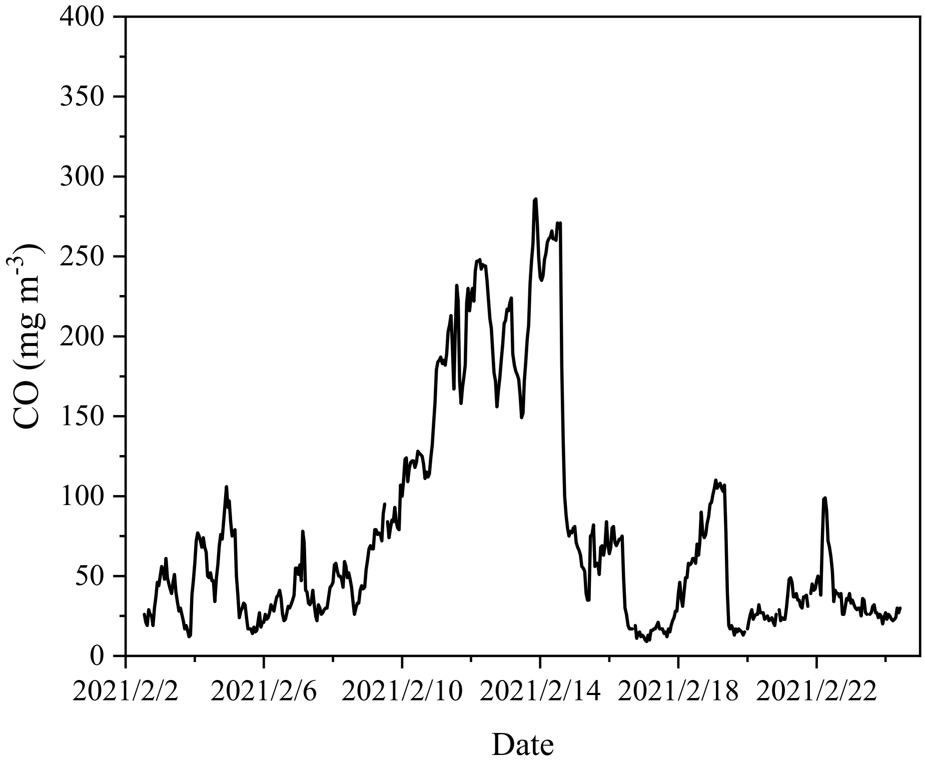

Supplementary Figure 1. Changes in carbon monoxide (CO) during the observation period. Time series of CO during the observation period in SJZ.

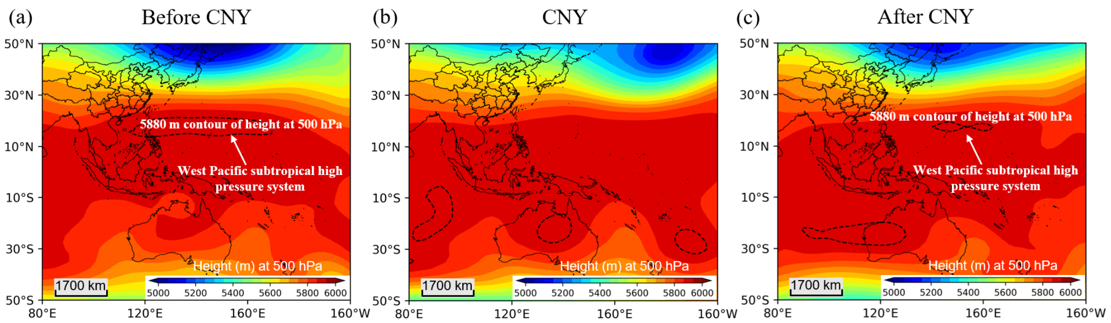

Supplementary Figure 2. Anomalous weakening of the western Pacific subtropical high-pressure system during the observation period. The West Pacific subtropical high-pressure system before CNY (**a**), during CNY (**b**), and after CNY (**c**) at 500 hPa.

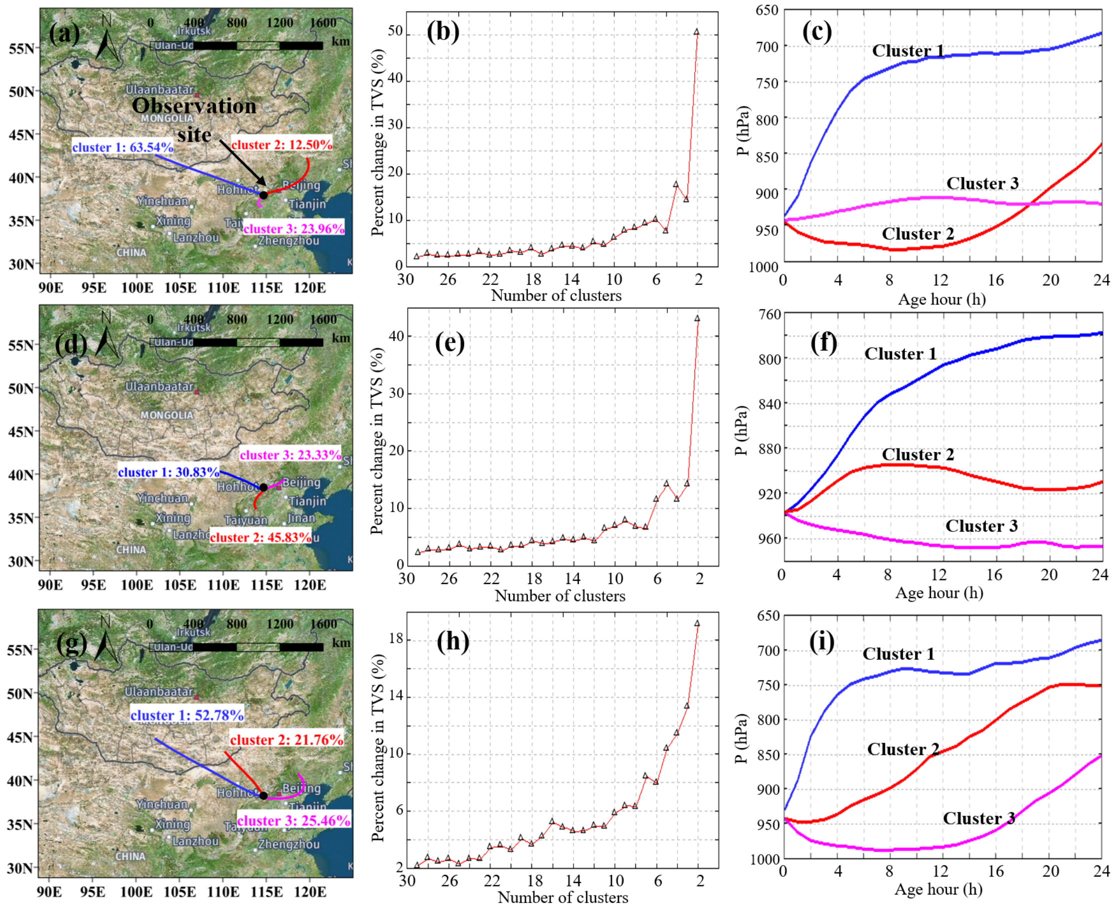

Supplementary Figure 3. Changes in backward trajectory clustering analysis during the observation period. The results of the cluster (**a**, **d**, **g**), the variation of total variation of spatial (TVS) (**b**, **e**, **h**), and aged mass pressure (**c**, **f**, **i**) during three periods in SJZ. (**a-c**: before CNY; **d-f**: CNY; **g-i**: after CNY. The 24-hour backward trajectories of air mass are at an altitude of 0.5 km)

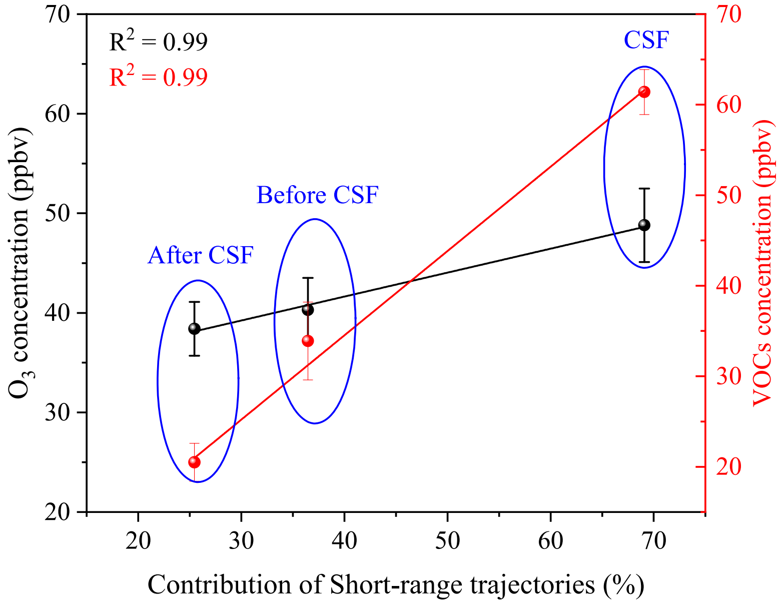

Supplementary Figure 4. The relationship between the percentage of short trajectories and the concentrations of O_3 and VOCs during the observation period. A high proportion of short trajectories occurs at high O_3 and VOC concentrations during CNY. Error bars for both O_3 and VOCs were calculated using standard deviation.

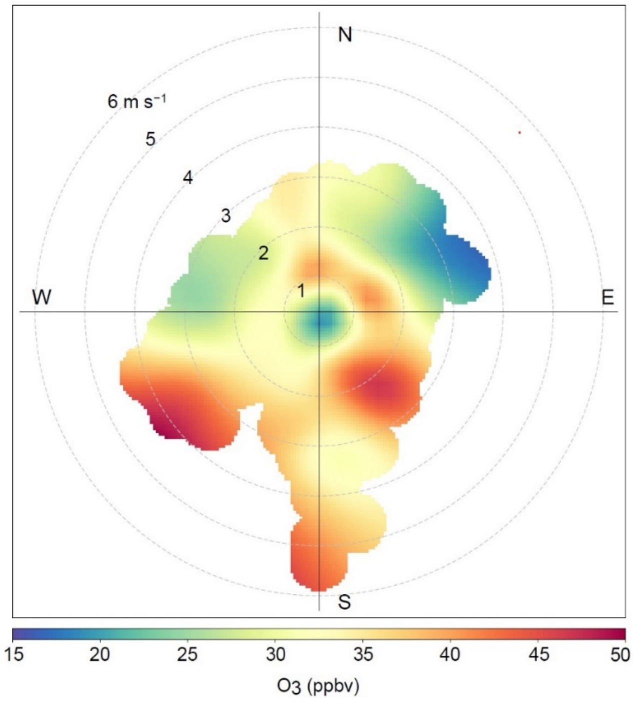

Supplementary Figure 5. Ozone distribution at different wind speeds and directions during the observation period. The relationship between WS&WD and O_3 during the observation.

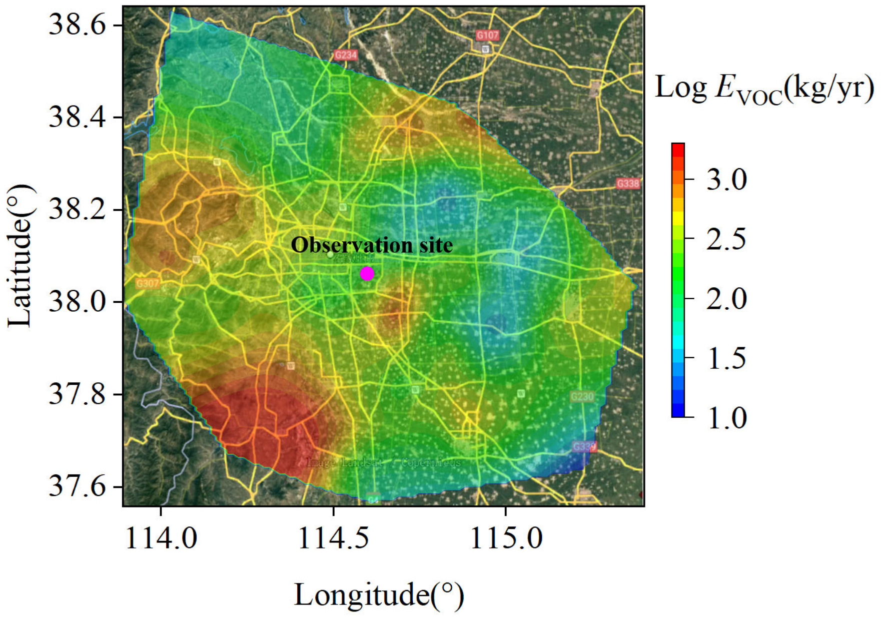

Supplementary Figure 6. VOC emissions in Shijiazhuang. Distribution of VOC emission and emission capacity in Shijiazhuang. The data were collected in 2020.

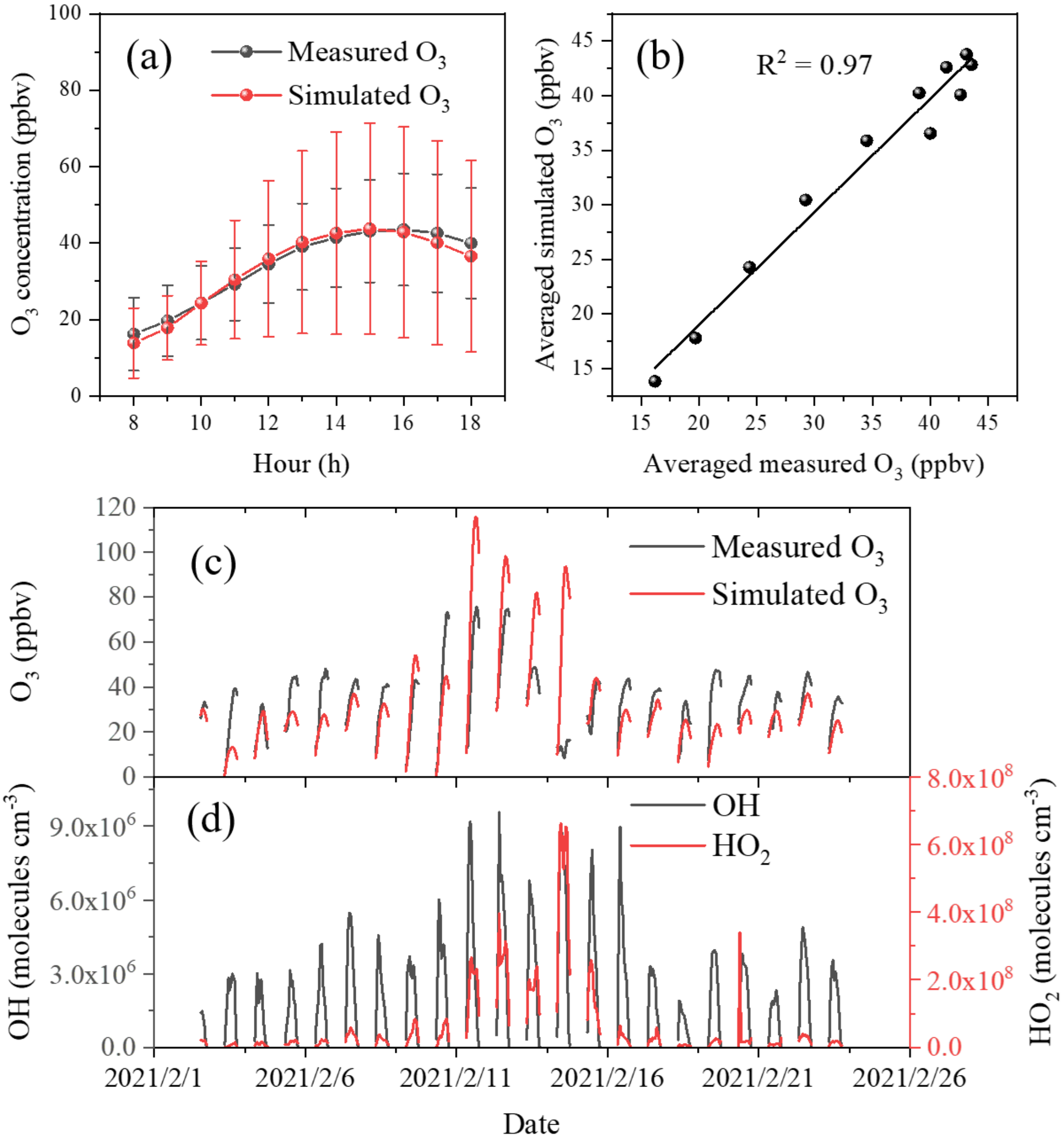

Supplementary Figure 7. Simulated performance of OBM. a represents the diurnal variation of observed and simulated O_3. Error bars for both observed and simulated O_3 were calculated using standard deviation. **b** represents the correlation between the average observed O_3 and average simulated O_3 during the daytime (8:00-18:00). **c** represents the comparison of observed and simulated O_3. **d** represents the simulated OH and HO_2 during the observation period.

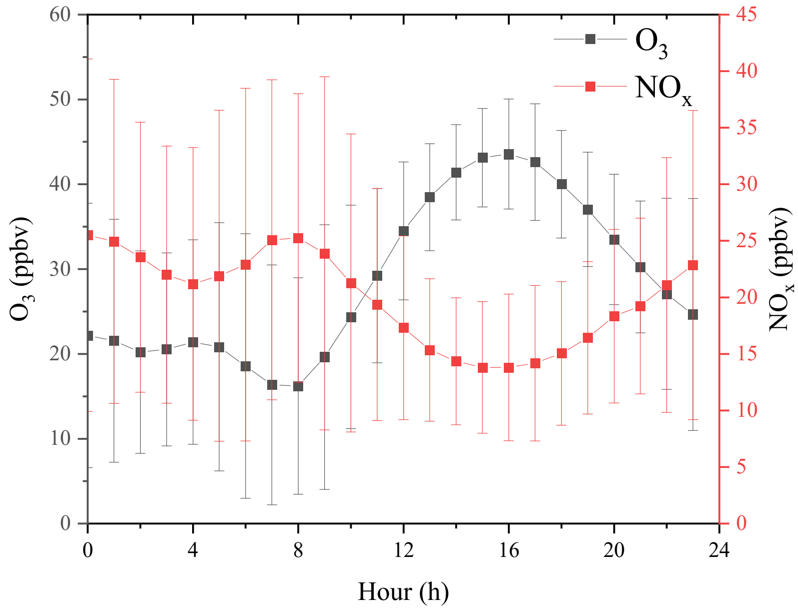

Supplementary Figure 8. Diurnal variation of O_3 and NO_x concentrations during the observation period. The black line indicates O_3 and the red line indicates NO_x. Error bars for both O_3 and NO_x were calculated using standard deviation.

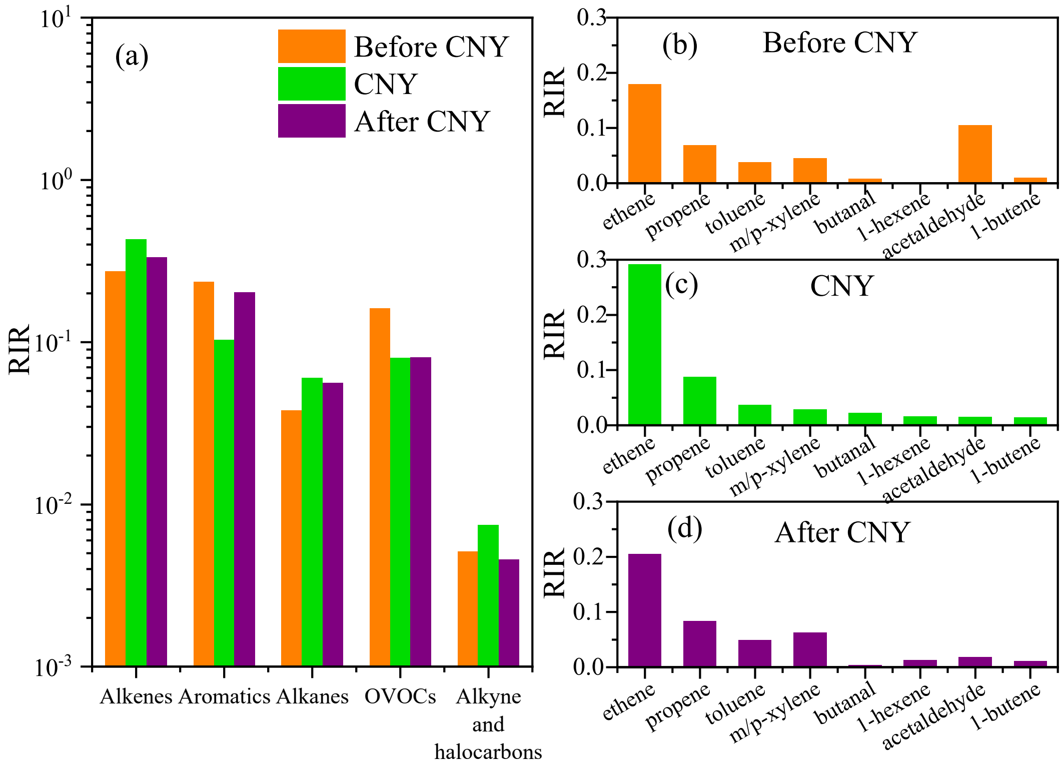

Supplementary Figure 9. The RIR values for different types and kinds of VOC. a RIR values for different types of VOCs. **b** RIR values for VOC species before CNY. c RIR values for VOC species during CNY. **d** RIR values for VOC species after CNY.

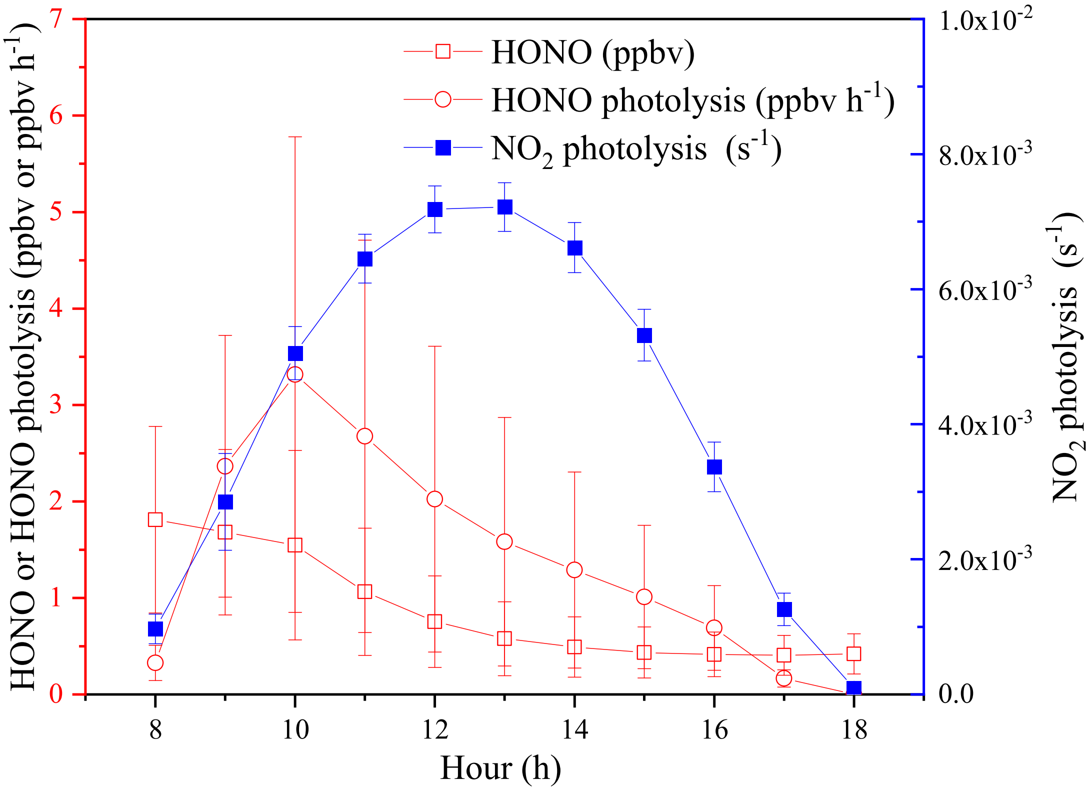

Supplementary Figure 10. The averaged HONO photolysis rate, HONO concentration and NO_2 photolysis rate during the observation. The red line indicates HONO and HONO photolysis and the blue line indicates NO_2 photolysis. Error bars for HONO photolysis rate, HONO concentration and NO_2 photolysis rate were calculated using standard deviation.

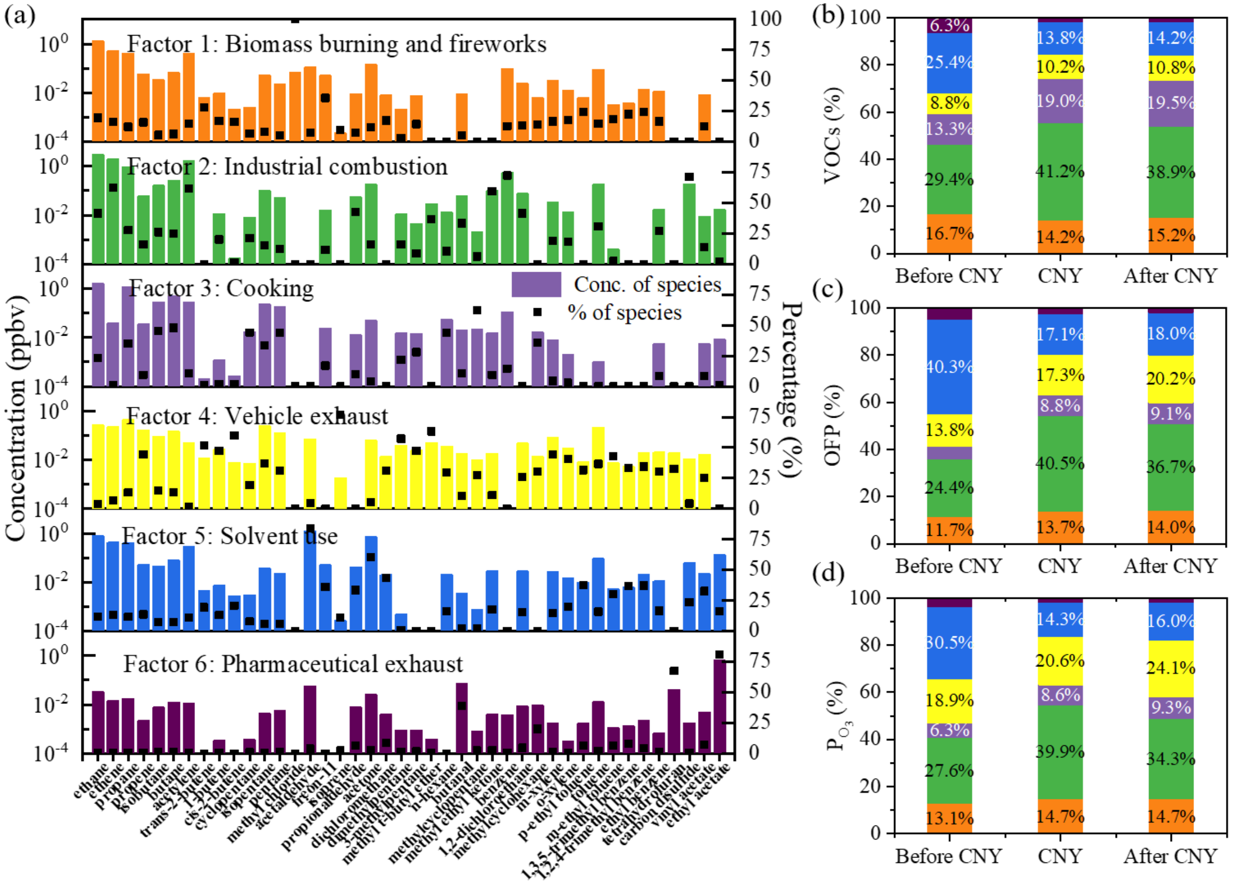

Supplementary Figure 11. Source profiles of VOCs and O_3 production rates of different emission sources during the wintertime observation period. Concentrations and percentages of the VOC sources (**a**), percentage of VOC (**b**), OFP (**c**), and O_3 production rate (**d**) for six factors.

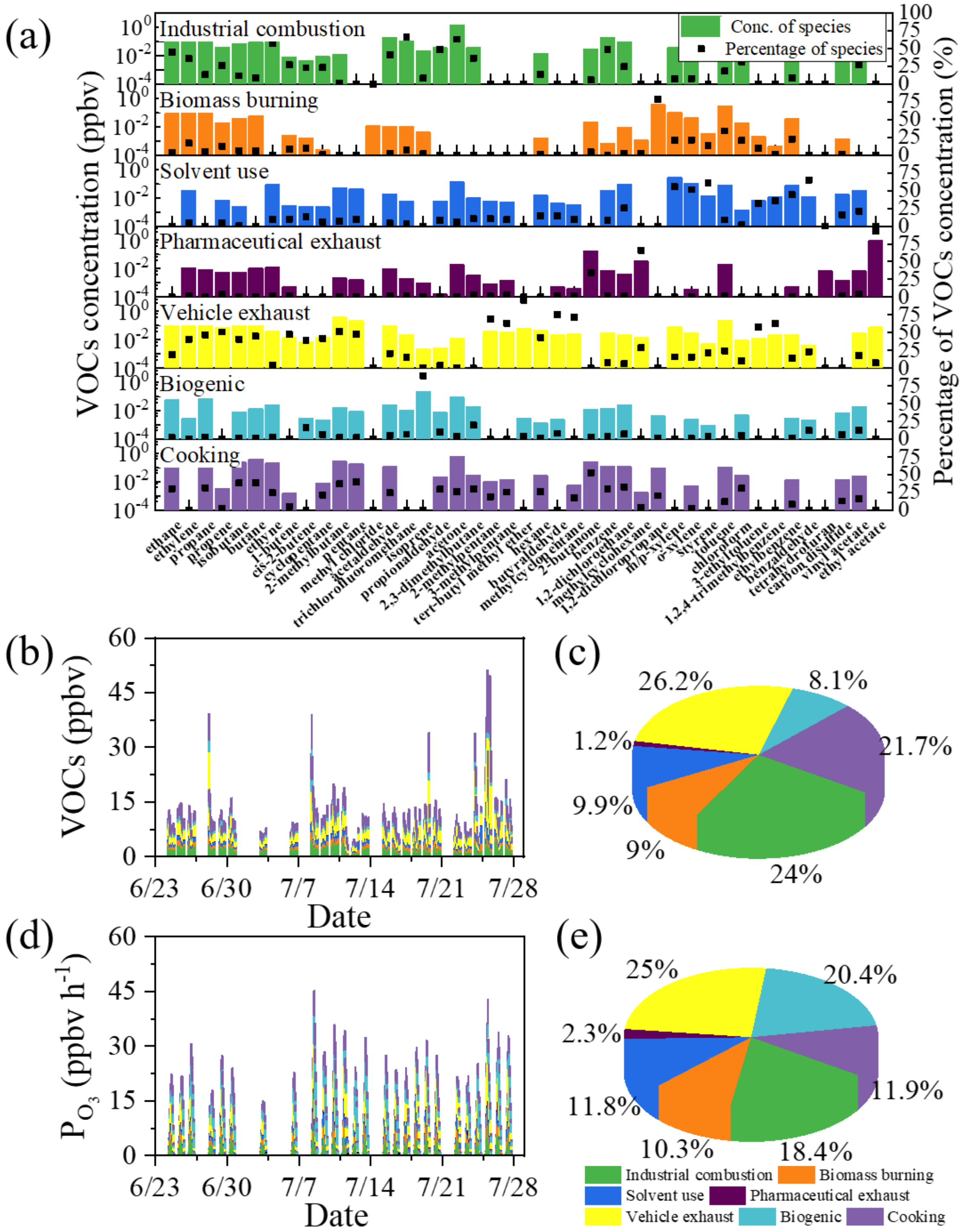

Supplementary Figure 12. Source profiles and O_3 production rates of different emission sources during the summertime observation period (June 24 to July 27, 2021). Concentrations and percentages of the VOCs during the summertime period (**a**), time series and percentage of VOCs (**b**, **c**) and P_{O3} (**d**, **e**) for seven factors. The observation sites and instruments are the same for winter and summer.

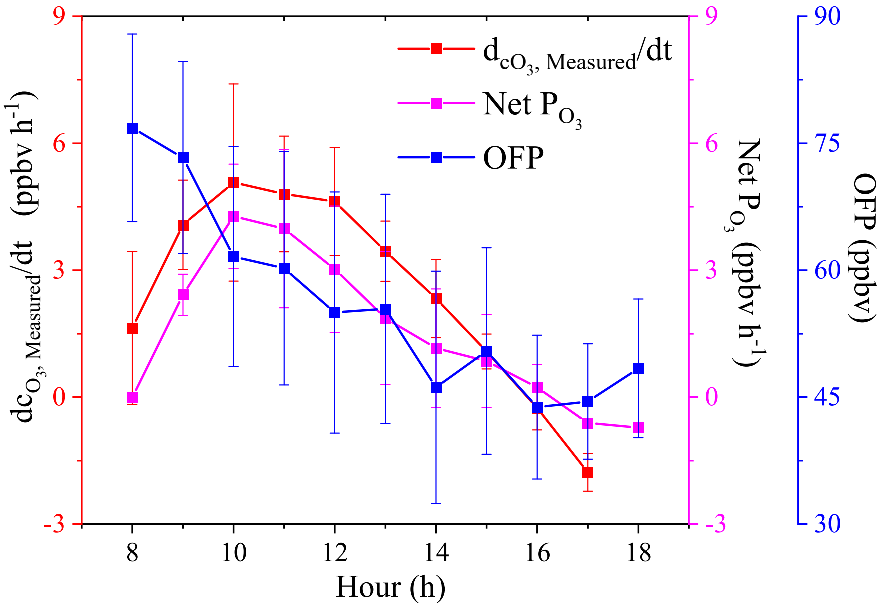

Supplementary Figure 13. The mean diurnal variation of change rates of measured O_3 concentrations, net P_{O3}, and OFP during the observation period. The red line shows the observed ozone change rate, the pink line shows the net rate of ozone production, and the blue line shows the OFP. Error bars were calculated using standard deviation.

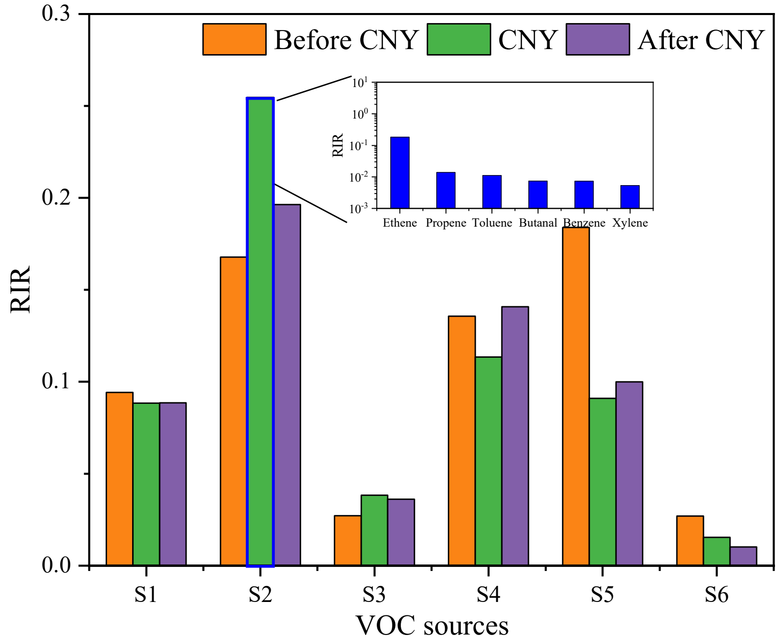

Supplementary Figure 14. Variation in RIR values for different sources. The RIR values for six factors before, during and after CNY. S1 refers to Biomass burning/fireworks. S2 refers to Industrial combustion. S3 refers to Cooking. S4 refers

to Vehicle exhaust. S5 refers to Solvent use. S6 refers to Pharmaceutical exhaust.

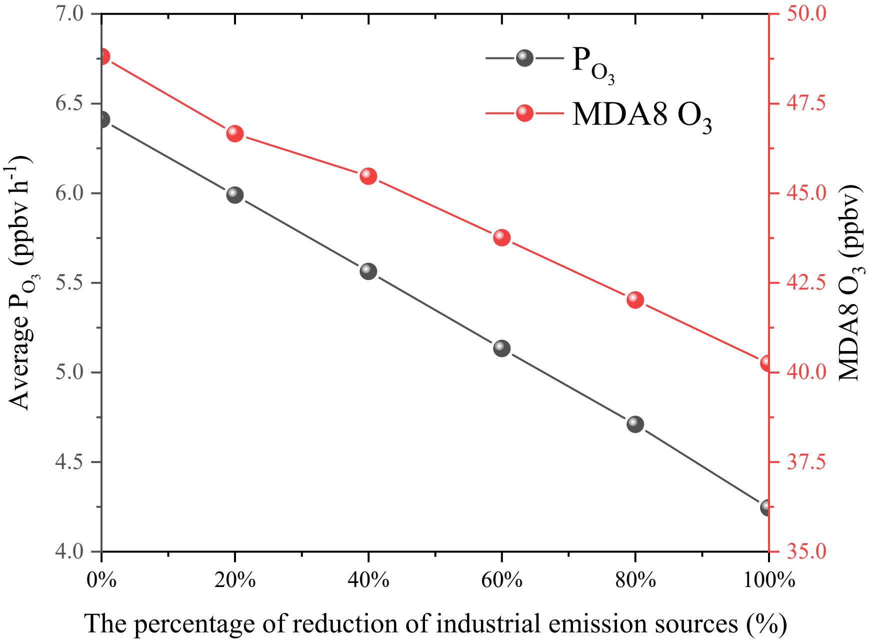

Supplementary Figure 15. Simulation of emission reduction from industrial sources under different scenarios. The P_{O3} and MDA8 O_3 under different reduction scenarios of industrial emission during CNY. The black line indicates P_{O3} and the red line indicates MDA8 O_3.

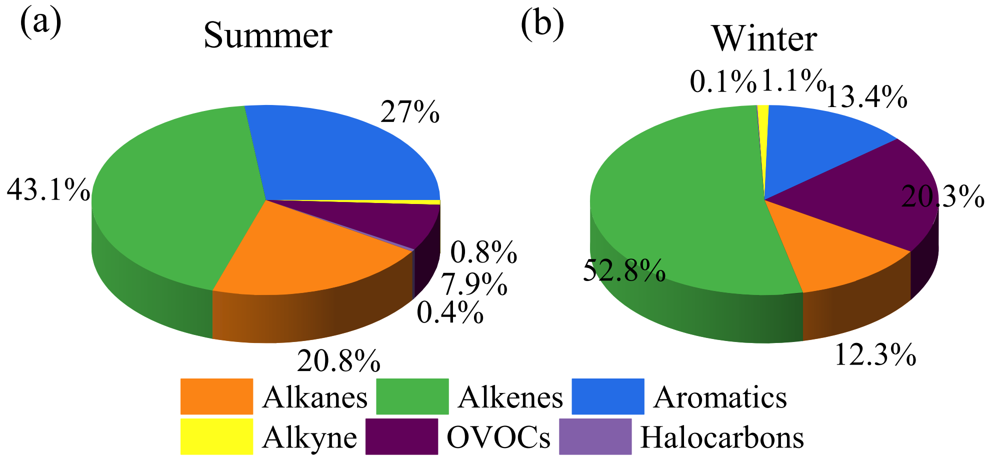

Supplementary Figure 16. Comparison of P_{O3} for different types of VOC in summer and winter. a Percentage of P_{O3} for six different types of VOC in summer (June 24 to July 27, 2021). **b** Percentage of P_{O3} for six different types of VOC in winter

(February 2 to 24, 2021).

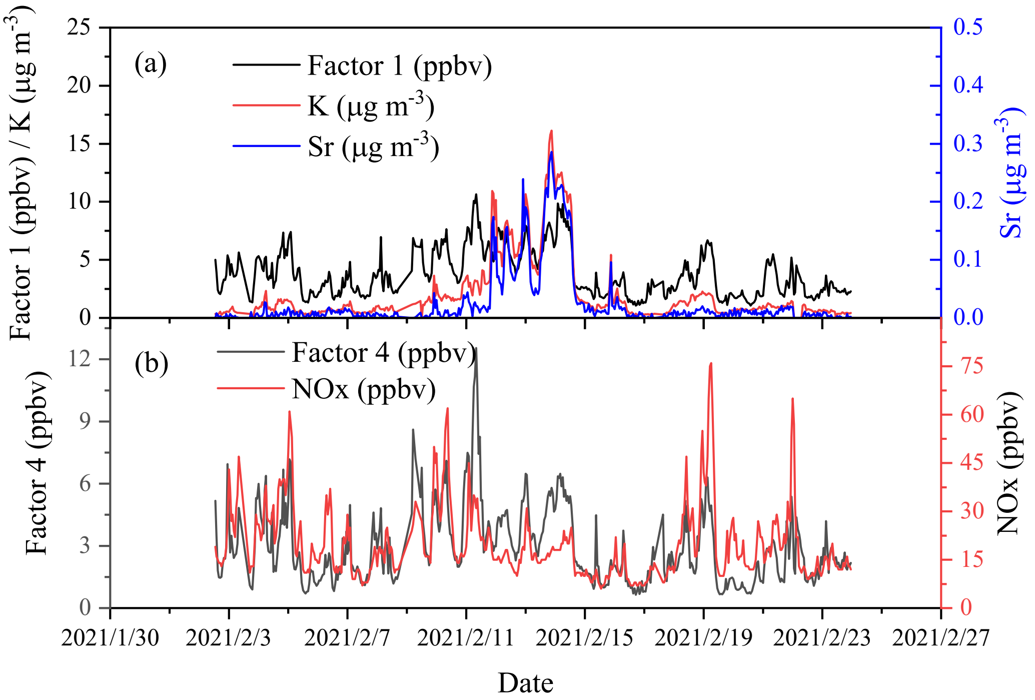

Supplementary Figure 17. **Changes in air pollutants during the observation period.** The time series of Factor 1, K and Sr (**a**), and the time series of Factor 4 and NO_x (**b**) during the observation.

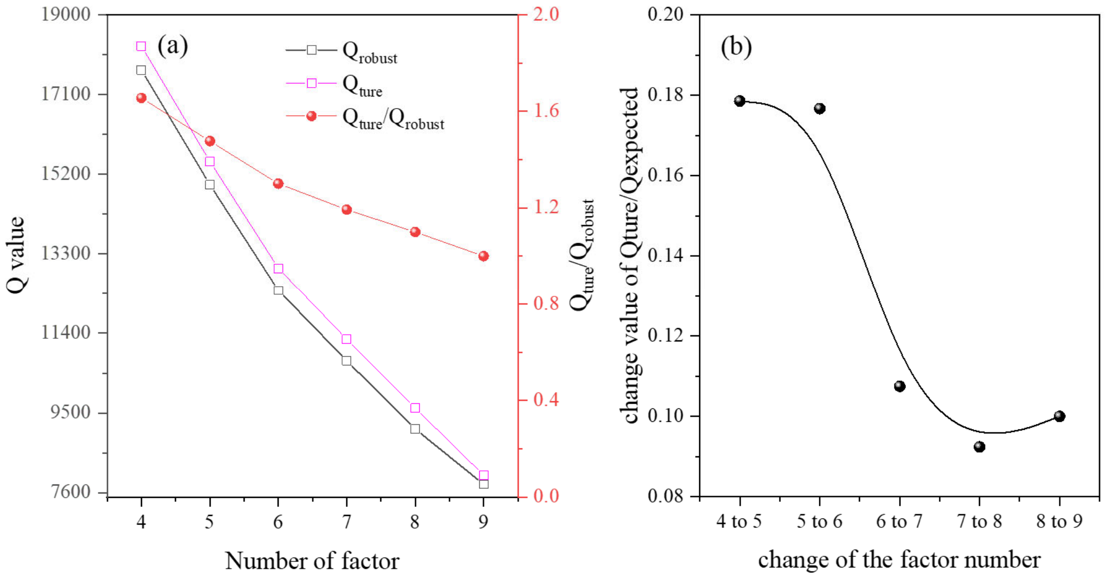

Supplementary Figure 18. **Variation of Q values in PMF simulations.** The relationship between factor number and Q value (**a**), and the relationship between the change of factor number and Qture/Qexpected (b).

Supplementary Figures

Supplementary Table 1. The different types of VOC and NO_x concentrations during the observation period. Concentrations of eight different types of pollutants in winter and summer. Unit: ppbv and the values were in the form of mean value ± standard deviation.

	Before CNY	CNY	After CNY	Summer (6/23 - 7/28)
Alkanes	10.6 ± 5.0	23.3 ± 7.3	10.6 ± 5.2	9.0 ± 5.5
Alkenes	3.5 ± 2.3	7.3 ± 3.6	3.0 ± 2.0	1.3 ± 0.7
Halocarbons	4.2 ± 9.9	1.0 ± 0.8	0.7 ± 0.7	1.7 ±0 .9
Alkynes	2.0 ± 1.1	5.2 ± 1.5	1.8 ± 0.9	0.9 ±0 .4
Aromatics	2.3 ± 1.8	3.5 ± 1.7	1.5 ± 1.1	2.3 ± 1.7
OVOC	10.9 ± 10.5	4.2 ± 2.2	2.7 ± 4.5	5.3 ± 5.4
CS_2	0.4 ± 1.0	0.5 ± 0.3	0.2 ± 0.3	0.2 ± 0.4
NO_x	21.9 ± 11.3	20.9 ± 9.9	17.5 ± 12.1	9.6 ± 5.1

Supplementary Table 2. The average percentage of VOC (species in both OBM and PMF), OFP, and O_3 production rate (P_{O3}) of six factors during the observation. Concentrations, OFP and P_{O3} change over different periods for six different emission sources.

Factor	Before CNY						CNY						After CNY					
	Conc.		OFP		P_{O3}		Conc.		OFP		P_{O3}		Conc.		OFP		P_{O3}	
	ppbv	%	ppbv	%	ppbv h^{-1}	%	ppbv	%	ppbv	%	ppbv h^{-1}	%	ppbv	%	ppbv	%	ppbv h^{-1}	%
Biomass burning and fireworks	6.4 ± 9.1	16.7± 12.6	7.5 ± 5.1	**11.7 ± 1.5**	0.3 ± 0.3	**13.1 ± 1.6**	6.1 ± 1.9	14.2 ± 0.7	11.2 ± 5.1	**13.7 ± 0.5**	0.9 ± 0.8	**14.7 ± 0.6**	2.8 ± 1.3	15.2 ± 1.5	5.0 ± 3.9	**14.0 ± 1.1**	0.2 ± 0.2	**14.7 ± 0.9**
Industrial combustion	9.0 ± 4.4	29.4 ± 6.5	15.3 ± 9.4	**24.4 ± 5.5**	0.6 ± 0.6	**27.6 ± 4.7**	17.8 ± 5.6	41.2 ± 2.6	33.0± 14.3	**40.5 ± 3.7**	2.6 ± 2.2	**39.9 ± 3.7**	7.4 ± 3.5	38.9 ± 3.1	12.4 ± 7.6	**36.7 ± 6.7**	0.5 ± 0.4	**34.3 ± 6.0**
Cooking	3.9 ± 1.8	13.3 ± 3.2	3.1 ± 1.8	**5.1 ± 0.8**	0.1 ± 0.1	**6.3 ± 1.2**	8.2 ± 2.6	19.0 ± 1.8	7.0 ± 3.0	**8.8 ± 1.3**	0.6 ± 0.5	**8.6 ± 1.6**	3.7 ± 1.8	19.5 ± 2.6	2.9 ± 1.3	**9.1 ± 2.6**	0.1 ± 0.1	**9.3 ± 2.4**
Vehicle exhaust	2.8 ± 1.6	8.8 ± 1.9	9.2 ± 7.4	**13.8 ± 2.7**	0.4 ± 0.4	**18.9 ± 5.5**	4.5 ± 2.0	10.2 ± 1.6	14.5 ± 8.8	**17.3 ± 2.9**	1.3 ± 1.3	**20.6 ± 3.6**	2.1 ± 1.2	10.8 ± 2.0	7.4 ± 6.9	**20.2 ± 4.4**	0.3 ± 0.3	**24.1 ± 5.2**
Solvent use	7.5 ± 3.2	25.4 ± 5.1	24.1± 12.3	**40.3 ± 6.0**	0.7 ± 0.7	**30.5± 7.9**	5.9 ± 1.8	13.8 ± 0.6	13.6 ± 5.5	**17.1 ± 1.1**	0.9 ± 0.8	**14.3 ± 1.0**	2.6 ± 1.2	14.2 ± 1.0	6.3 ± 5.1	**18.0 ± 2.2**	0.2 ± 0.2	**16.0 ± 2.2**
Pharmaceutical exhaust	2.8 ± 6.3	6.3 ± 7.7	3.2 ± 3.4	**4.7 ± 3.2**	0.1 ± 0.2	**3.6 ± 2.7**	0.8 ± 0.7	1.7 ± 1.2	2.1 ± 1.2	**2.6 ± 1.0**	0.1 ± 0.1	**1.9 ± 1.0**	0.3 ± 0.6	1.5 ± 1.9	0.8 ± 1.4	**1.9 ± 1.0**	0.03 ± 0.0	**1.7 ± 1.2**
Sum	32.4	100	62.4	**100**	2.2	**100**	43.3	100	81.4	**100**	6.4	**100**	18.9	100	35.0	**100**	1.4	**100**

Supplementary Table 3. Cross-testing of weather conditions during CNY in Shijiazhuang. Comparison of P_{O3} for different emission sources under meteorological conditions in summer and winter. Meteorological conditions include temperature, humidity, atmospheric pressure, and photolysis rate constants.

Factor	Meteorological (winter)+VOCs (winter, CNY)		Meteorological (summer)+VOCs (winter, CNY)	
	Value / ppbv h^{-1}	Percentage / %	Value / ppbv h^{-1}	Percentage / %
Biomass burning and fireworks	0.9 ± 0.8	14.7 ± 0.6	1.7 ± 1.5	13.5 ± 1.1
Industrial combustion	2.6 ± 2.2	**39.9 ± 3.7**	4.1 ± 3.6	**32.8 ± 3.8**
Cooking	0.6 ± 0.5	8.6 ± 1.6	0.9 ± 0.8	7.5 ± 0.8
Vehicle exhaust	1.3 ± 1.3	20.6 ± 3.6	2.6 ± 2.4	20.4 ± 3.9
Solvent use	0.9 ± 0.8	14.3 ± 1.0	2.9 ± 2.3	22.7 ± 4.5
Pharmaceutical exhaust	0.1 ± 0.1	1.9 ± 1.0	0.4 ± 0.3	3.2 ± 1.1

Supplementary Table 4. Details of the instruments used in this study. Different instrument models, manufacturers, and measured pollutants are included.

Instrument	Model	Manufacturer	Country	Target pollutant
GC-MS/FID	EXPEC2000-MS	Expec-Tech.	China	VOCs
trace gas analyzer	42i, 43i, 48i, and 49i	Thermo Scientific	USA	NOx, SO_2, CO, O_3
MARGA	ADI 2080	Applikon Analytical B.V.	Netherlands	HONO (g), K^+ (l), Cl^- (l)
$PM_{2.5}$	BAM-1020	Met One Instruments	USA	$PM_{2.5}$
weather station	WXT 520	Vaisala	Finland	temperature (T), pressure (P), relative humidity (RH), wind speed (WS), wind direction (WD)

Supplementary Table 5. Average concentration, standard deviation, method detection limit (MDL), and relative standard derivation (RSD) of different VOC species during the observation.

Species	Average[1]	Standard deviation[1]	MDL[1]	RSD[2]	Species	Average[1]	Standard deviation[1]	MDL[1]	RSD[2]
**Alkanes**					1,2,3-trimethylbenzene[†]	0.01	0.07	0.06	4.06
ethane[†, ‡]	6.92	3.71	0.04	2.00	4-ethyltoluene[†, ‡]	0.02	0.08	0.08	5.87
propane[†, ‡]	3.49	2.11	0.01	0.00	2-ethyltoluene[†]	0.01	0.03	0.03	2.01
n-butane[†, ‡]	1.07	0.69	0.03	1.99	Cumene[†]	0.01	0.03	0.05	3.99
n-pentane[†, ‡]	0.42	0.37	0.03	1.96	propyl benzene[†]	0.01	0.04	0.04	2.01
i-butane[†, ‡]	0.71	0.66	0.02	1.98	**OVOCs**				
n-hexane[†, ‡]	0.13	0.11	0.05	4.03	butyraldehyde[†, ‡]	0.25	0.73	0.08	3.79
i-pentane[†, ‡]	0.71	0.66	0.02	1.99	tetrahydrofuran[‡]	0.10	0.34	0.06	5.97
cyclopentane[‡]	0.04	0.03	0.02	1.98	vinyl acetate[‡]	0.07	0.09	0.05	3.97
methylcyclopentane[‡]	0.03	0.03	0.07	6.36	acetaldehyde[†, ‡]	1.53	1.77	0.07	3.64
methylcyclohexane[‡]	0.04	0.08	0.06	3.99	valeraldehyde[†]	0.17	0.19	0.08	5.77
2-methylpentane[†, ‡]	0.07	0.07	0.05	5.93	propionaldehyde[†, ‡]	0.21	0.24	0.09	5.85
2-methylhexane[†]	0.08	0.06	0.06	3.97	acrolein[†]	0.16	0.28	0.08	5.80

3-methylpentane†, ‡	0.05	0.05	0.03	3.99	2-butanone†, ‡	0.22	0.28	0.05	3.91
2,3-dimethylbutane†	0.02	0.03	0.05	5.77	methacrolein†	0.02	0.03	0.07	3.91
3-methylhexane†	0.02	0.02	0.03	2.02	acetone†, ‡	1.22	0.95	0.09	5.98
2,2-dimethylbutane†	0.01	0.01	0.05	4.07	4-methyl-2-pentanone†	0.02	0.11	0.05	3.98
n-octane†	0.00	0.01	0.04	1.98	hexanal†	0.00	0.01	0.09	5.71
n-nonane†	0.01	0.05	0.05	4.03	2-hexanone†	0.00	0.02	0.07	4.01
decane†	0.03	0.10	0.07	4.01	3-methyl benzaldehyde†	0.03	0.20	0.08	5.93
n-hendecane†	0.04	0.12	0.09	5.92	benzaldehyde†	0.05	0.20	0.05	3.89
dodecane†	0.05	0.28	0.08	6.02	methyl tert-butyl ether†, ‡	0.07	0.11	0.08	5.95
**Alkyne**					ethyl acetate†, ‡	0.93	4.10	0.07	3.95
acetylene†, ‡	2.71	1.84	0.05	3.92	isopropyl alcohol†	0.70	2.74	0.07	3.98
**Alkenes**					**Halo hydrocarbons**				
ethene†, ‡	3.66	2.86	0.09	6.16	1,2-dichloropropane†	0.04	0.07	0.05	4.01
propene†, ‡	0.41	0.30	0.02	1.97	trichloroethylene†	0.00	0.01	0.05	4.05
1-hexene†	0.05	0.06	0.07	4.00	methylchloride†, ‡	1.02	5.17	0.10	6.31
1-butene†, ‡	0.06	0.05	0.02	1.97	freon-11‡	0.14	0.04	0.05	3.98

1,3-butadiene[†]	0.03	0.04	0.08	6.10
trans-2-pentene[†]	0.00	0.01	0.03	3.83
cis-2-pentene[†]	0.00	0.01	0.04	3.44
trans-2-butene[‡]	0.02	0.02	0.03	1.99
cis-2-butene[‡]	0.01	0.01	0.02	2.01
1-pentene[†]	0.02	0.03	0.06	6.52
isoprene[†, ‡]	0.002	0.01	0.07	5.66
**Aromatics**				
m/p-xylene[†, ‡]	0.21	0.18	0.09	3.05
toluene[†, ‡]	0.80	0.87	0.09	2.03
1,2,4-trimethylbenzene[†, ‡]	0.05	0.12	0.05	4.06
o-xylene[†, ‡]	0.08	0.06	0.05	4.06
benzene[†, ‡]	0.93	0.71	0.04	2.05
ethylbenzene[†, ‡]	0.07	0.06	0.05	4.06
1,3,5-trimethylbenzene[†, ‡]	0.02	0.09	0.06	3.97
3-ethyltoluene[†, ‡]	0.02	0.04	0.03	1.92
1,2-dichloroethane[‡]	0.43	0.73	0.07	4.01
dichloromethane[†]	0.09	0.31	0.08	6.07
chloroform[†]	0.04	0.05	0.04	2.00
chloroethylene[†]	0.04	0.05	0.05	3.93
methyl bromide[†]	0.08	0.38	0.06	3.91
chloroethane[†]	0.00	0.00	0.08	5.83
1,1-dichloroethane[†]	0.00	0.01	0.07	4.00
1,1,1-trichloroethane[†,]	0.00	0.01	0.04	2.00
1,1,2-trichloroethane[†]	0.00	0.00	0.09	5.54
tetrachloroethylene[†]	0.00	0.01	0.08	6.02
1,2-dibromoethane[†]	0.00	0.00	0.03	1.99
1,1,2,2-tetrachloroethane[†]	0.00	0.02	0.05	4.03
**Other**				
carbon disulfide[‡]	0.34	0.63	0.08	5.89

styrene[†, ‡]	0.02	0.06	0.05	4.06

[1] **Unit:** ppbv.

[2] **Unit:** %.

[†] **Note:** This species was used in the OBM.

[‡] **Note:** This species was used in the PMF.

Supplementary Table 6. BS mapping for a six-factor solution. S1 refers to Biomass burning/fireworks. S2 refers to Industrial combustion. S3 refers to Cooking. S4 refers to Vehicle exhaust. S5 refers to Solvent use. S6 refers to Pharmaceutical exhaust.

	S1	S2	S3	S4	S5	S6	Unmapped
Boot S1	87	8	1	0	4	0	0
Boot S2	2	98	0	0	0	0	0
Boot S3	1	6	90	2	1	0	0
Boot S4	0	1	0	99	0	0	0
Boot S5	1	9	1	3	86	0	0
Boot S6	0	1	0	0	0	99	0

Supplementary References

1. Liu, Y., Shao, M., Fu, L., Lu, S., Zeng, L., *et al.* Source profiles of volatile organic compounds (VOCs) measured in China: Part I. *Atmos. Environ.* **42**, 6247–6260 (2008).
2. Kim, B. M., Seo, J., Kim, J. Y., Lee, J. Y. & Kim, Y. Transported vs. local contributions from secondary and biomass burning sources to $PM_{2.5}$. *Atmos. Environ.* **144**, 24–36 (2016).
3. Srivastava, D., Xu, J., Vu, T. V., Liu, D., Li, L., *et al.* Insight into $PM_{2.5}$ sources by applying positive matrix factorization (PMF) at urban and rural sites of Beijing. *Atmos. Chem. Phys.* **21**, 14703–14724 (2021).
4. Lin, C.-C. A review of the impact of fireworks on particulate matter in ambient air. *J. Air Waste Manag. Assoc.* **66**, 1171–1182 (2016).
5. Rindelaub, J. D., Davy, P. K., Talbot, N., Pattinson, W. & Miskelly, G. M. The contribution of commercial fireworks to both local and personal air quality in Auckland, New Zealand. *Environ. Sci. Pollut. Res.* **28**, 21650–21660 (2021).
6. Xiao, Y., Jacob, D. J. & Turquety, S. Atmospheric acetylene and its relationship with CO as an indicator of air mass age. *J. Geophys. Res. Atmospheres* **112**, (2007).
7. Chen, W. T., Shao, M., Lu, S. H., Wang, M., Zeng, L. M., *et al.* Understanding primary and secondary sources of ambient carbonyl compounds in Beijing using the PMF model. *Atmos. Chem. Phys.* **14**, 3047–3062 (2014).
8. Karan, K. & Behie, L. A. CS_2 Formation in the Claus Reaction Furnace: A Kinetic Study of Methane−Sulfur and Methane−Hydrogen Sulfide Reactions. *Ind. Eng. Chem. Res.* **43**, 3304–3313 (2004).
9. Russo, R. S., Zhou, Y., White, M. L., Mao, H., Talbot, R., *et al.* Multi-year (2004–2008) record of nonmethane hydrocarbons and halocarbons in New England: seasonal variations and regional sources. *Atmos. Chem. Phys.* **10**, 4909–4929 (2010).
10. Cheng, S., Wang, G., Lang, J., Wen, W., Wang, X., *et al.* Characterization of volatile organic compounds from different cooking emissions. *Atmos. Environ.* **145**, 299–307 (2016).
11. Lyu, X., Wang, N., Guo, H., Xue, L., Jiang, F., *et al.* Causes of a continuous summertime O_3 pollution event in Jinan, a central city in the North China Plain. *Atmos. Chem. Phys.* **19**, 3025–3042 (2019).
12. Liu, X., Guo, H., Zeng, L., Lyu, X., Wang, Y., *et al.* Photochemical ozone pollution in five Chinese megacities in summer 2018. *Sci. Total Environ.* **801**, 149603 (2021).
13. McCarthy, M. C., Aklilu, Y.-A., Brown, S. G. & Lyder, D. A. Source apportionment of volatile organic compounds measured in Edmonton, Alberta. *Atmos. Environ.* **81**, 504–516 (2013).
14. Anenberg, S. C., Miller, J., Minjares, R., Du, L., Henze, D. K., *et al.* Impacts and mitigation of excess diesel-related NOx emissions in 11 major vehicle markets. *Nature* **545**, 467–471 (2017).
15. Cai, C., Geng, F., Tie, X., Yu, Q. & An, J. Characteristics and source apportionment of VOCs measured in Shanghai, China. *Atmos. Environ.* **44**, 5005–5014 (2010).
16. Mo, Z., Shao, M. & Lu, S. Compilation of a source profile database for hydrocarbon and

OVOC emissions in China. *Atmos. Environ.* **143**, 209–217 (2016).

17. Seila, R. L., Main, H. H., Arriaga, J. L., Martínez V, G. & Ramadan, A. B. Atmospheric volatile organic compound measurements during the 1996 Paso del Norte Ozone Study. *US-Mex. Transbound. Air Pollut. Stud.* **276**, 153–169 (2001).
18. Jensen, A., Liu, Z., Tan, W., Dix, B., Chen, T., *et al.* Measurements of Volatile Organic Compounds During the COVID-19 Lockdown in Changzhou, China. *Geophys. Res. Lett.* **48**, e2021GL095560 (2021).
19. Lin, Q., Gao, Z., Zhu, W., Chen, J. & An, T. Underestimated contribution of fugitive emission to VOCs in pharmaceutical industry based on pollution characteristics, odorous activity and health risk assessment. J. Environ. Sci. 126, 722-733 (2023).
20. Zhang, X., Li, H., Wang, X., Zhang, Y., Bi, F., *et al.* Heavy ozone pollution episodes in urban Beijing during the early summertime from 2014 to 2017: Implications for control strategy. *Environ. Pollut.* **285**, 117162 (2021).
21. Fan, X., Cai, J., Yan, C., Zhao, J., Guo, Y., *et al.* Atmospheric gaseous hydrochloric and hydrobromic acid in urban Beijing, China: detection, source identification and potential atmospheric impacts. *Atmos. Chem. Phys.* **21**, 11437–11452 (2021).
22. Liu, Y., Qiu, P., Li, C., Li, X., Ma, W., *et al.* Evolution and variations of atmospheric VOCs and O_3 photochemistry during a summer O_3 event in a county-level city, Southern China. *Atmos. Environ.* **272**, 118942 (2022).
23. Gao, J., Zhang, J., Li, H., Li, L., Xu, L., *et al.* Comparative study of volatile organic compounds in ambient air using observed mixing ratios and initial mixing ratios taking chemical loss into account – A case study in a typical urban area in Beijing. *Sci. Total Environ.* **628–629**, 791–804 (2018).
